# Supplementary material for: Synthesis of Novel Halogenated Heterocycles Based on o-Phenylenediamine and Their Interactions with the Catalytic Subunit of Protein Kinase CK2
Source: Molecules. 2021 May 25;26(11):3163. doi: 10.3390/molecules26113163 (PMC8198750; doi:10.3390/molecules26113163)
Supplement: Supplementary file 1 [file molecules-26-03163-s001.zip › molecules-1145649-supplementary.pdf]

Supplementary Materials for

Synthesis of novel halogenated heterocycles based on *o*-phenylenediamine and evaluation of its interactions with protein kinase CK2.

Maria Winiewska-Szajewska[1], Agnieszka M. Maciejewska[1], Elżbieta Speina[1], Jarosław Poznański[1*] and Daniel Paprocki[1*]

1 Institute of Biochemistry and Biophysics, Polish Academy of Sciences, Pawinskiego 5a, 02-106 Warsaw, Poland.

Table of contents

1. Chemistry 3

Synthesis of 4,5-diiodobenzene-1,2-diamine 3

1,2-diiodo-4,5-dinitrobenzene 3

1,2-diamino-4,5-diiodobenzene (1e) 4

Synthesis of 2,1,3-benzothiadiazole derivatives (2a-e) 4

General procedure: 4

2,1,3-benzothiadiazole (2a) 4

5,6-difluoro-2,1,3-benzothiadiazole (2b) 6

5,6-dichloro-2,1,3-benzothiadiazole (2c) 7

5,6-dibromo-2,1,3-benzothiadiazole (2d) 8

5,6-diiodo-2,1,3-benzothiadiazole (2e) 9

Synthesis of 2-trifluoromethylo-1*H*-benzimidazole derivatives (3a-e) 10

General procedure: 10

2-trifluoromethylo-1*H*-benzimidazole (3a) 11

5,6-difluoro-2-trifluoromethylo-1*H*-benzimidazole (3b) 12

5,6-dichlorlo-2-trifluoromethylo-1*H*-benzimidazole (3c) 12

5,6-dibromo-2-trifluoromethylo-1*H*-benzimidazole (3d) 13

5,6-diiodo-2-trifluoromethylo-1*H*-benzimidazole (3e) 13

Synthesis of 2-hydroxymethylo-1*H*-benzimidazole derivatives (4a,c-e) 14

General procedure: 14

2-hydroxymethylo-1*H*-benzimidazole (4a) 15

5,6-dichloro-2-hydroxymethylo-1*H*-benzimidazole (4c) 16

5,6-dibromo-2-hydroxymethylo-1*H*-benzimidazole (4d) 17

5,6-diiodo-2-hydroxymethylo-1*H*-benzimidazole (4e) 18
Synthesis of 1*H*-benzo[d]imidazol-2(3*H*)-one derivatives (5a,c-e) 19
General procedure: 19
1*H*-benzo[d]imidazol-2(3*H*)-one (5a) 20
5,6-dichloro-1*H*-benzo[d]imidazol-2(3*H*)-one (5c) 21
5,6-dibromo-1*H*-benzo[d]imidazol-2(3*H*)-one (5d) 22
5,6-diiodo-1*H*-benzo[d]imidazol-2(3*H*)-one (5e) 23
Synthesis of quinoxaline derivatives (6a,c-e) 24
General procedure: 24
quinoxaline (6a) 25
6,7-dichloroquinoxaline (6c) 26
6,7-dibromoquinoxaline (6d) 27
6,7-diiodoquinoxaline (6e) 28
Synthesis of quinoxaline-2,3-diol derivatives (7a-e) 29
General procedure: 29
quinoxaline-1,3-diol (7a) 30
6,7-difluoroquinoxaline-1,3-diol (7b) 31
6,7-dichloroquinoxaline-1,3-diol (7c) 32
6,7-dibromooquinoxaline-1,3-diol (7d) 33
6,7-diiodooquinoxaline-1,3-diol (7e) 34
2. Cell viability 35
3. Enzymatic in vitro activity. 44
4. Molecular modeling data 45

1. Chemistry

Synthesis of 4,5-diiodobenzene-1,2-diamine

1,2-diiodo-4,5-dinitrobenzene

Iodine (3,5 g) was dissolved in oleum (10 mL of 20 % solution of SO_3 in H_2SO_4) and heated at 120 °C for 20 min. o-Dinitrobenzene (2.27 g, 13.5 mmol) was added. The resulting mixture was heated at 120 °C for 80 min. The reaction mixture was then poured into 500 mL of water with ice and NaOH was added until the solution was alkaline. It was extracted with chloroform (3x50 mL), combined organic phases were washed with saturated $Na_2S_2O_3$ solution (100 mL) and water (100 mL) and dried over $MgSO_4$, filtered and concentrated to dryness. Recrystallization from EtOH/H_2O gave 1,2-diiodo-4,5-dinitrobenzene, 3.01 g, yield = 53 %: mp 182-183 °C (lit.[3] mp 183-184 °C); ^{1}H NMR (500 MHz, d-DMSO) δ 8.64 (2H, s, Ar*H*); HRMS calcd. for $C_6H_5I_2N_2O_2$ $[M+H]^+$: 390.84349, found: 390.84325.

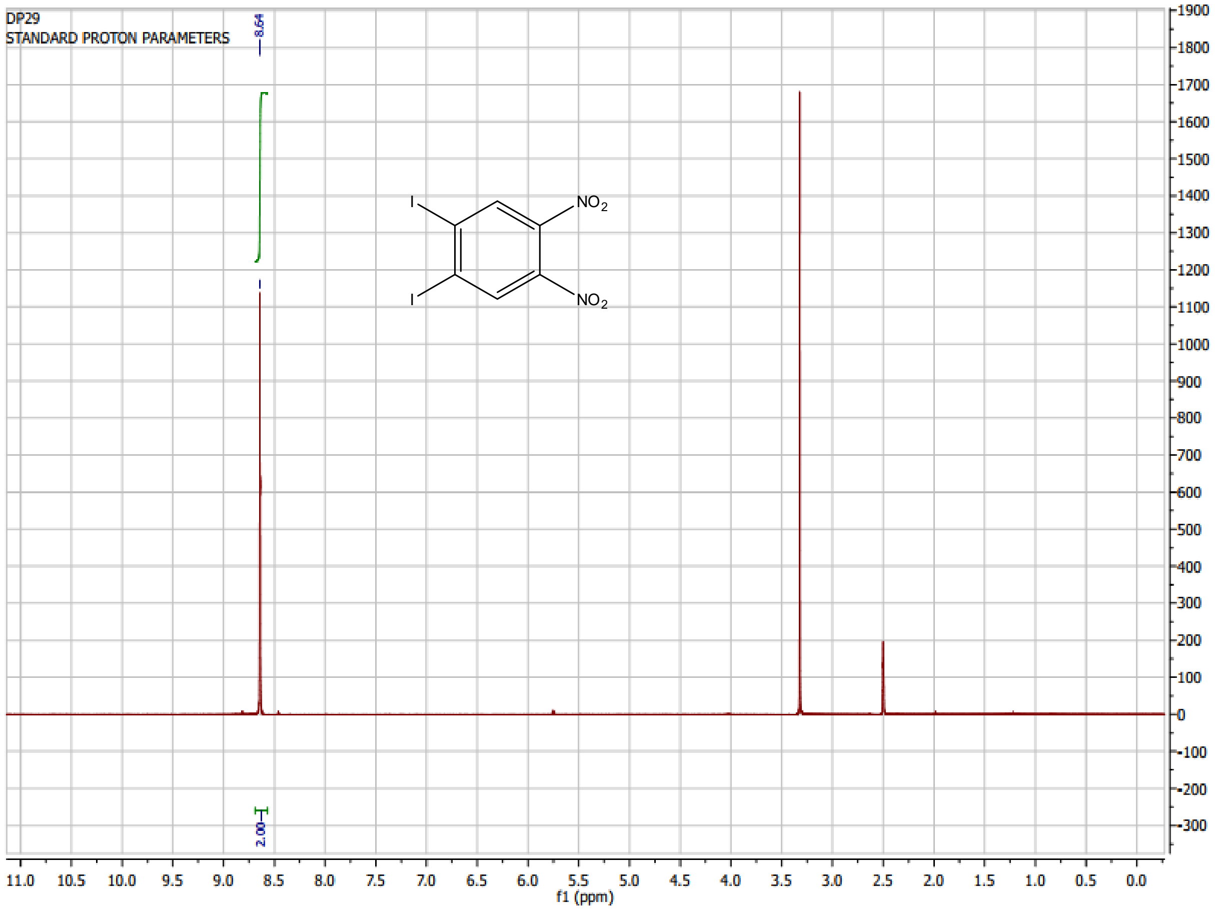

Figure S1. ^{1}H NMR spectrum of 1,2-diiodo-4,5-dinitrobenzene.

1,2-diamino-4,5-diiodobenzene (1e)

Tin(II) chloride dehydrate (47.6 mmol, 10.74 g) was dissolved concentrated HCl (50 mL) at 60 °C. 1,2-Diiodo-4,5-dinitrobenzene (4.76 mmol, 2 g) and conc. HCl (10 mL) were cooled in an ice-bath and a mixture of $SnCl_2x2H_20$ was added dropwise. The resulting reaction mixture was heated at 60 °C for 24 h. Afterwards, the reaction mixture was cooled to room temperature, filtered and washed with 2M HCl (3x5 mL) and ethyl ether (5 mL) giving 1,2-diamino-4,5-diiodobenzene. 1.63 g, yield = 95 %: mp 133-134 °C (lit. mp 135-136 °C); ^{1}H NMR (500 MHz, d-DMSO) δ 7.52 (2H, s, Ar*H*), 8.29 (4H, s br, NH_2+H_2O); HRMS calcd. for $C_6H_7I_2N_2$ [M+H]$^+$: 360.86931, found: 360.86925.

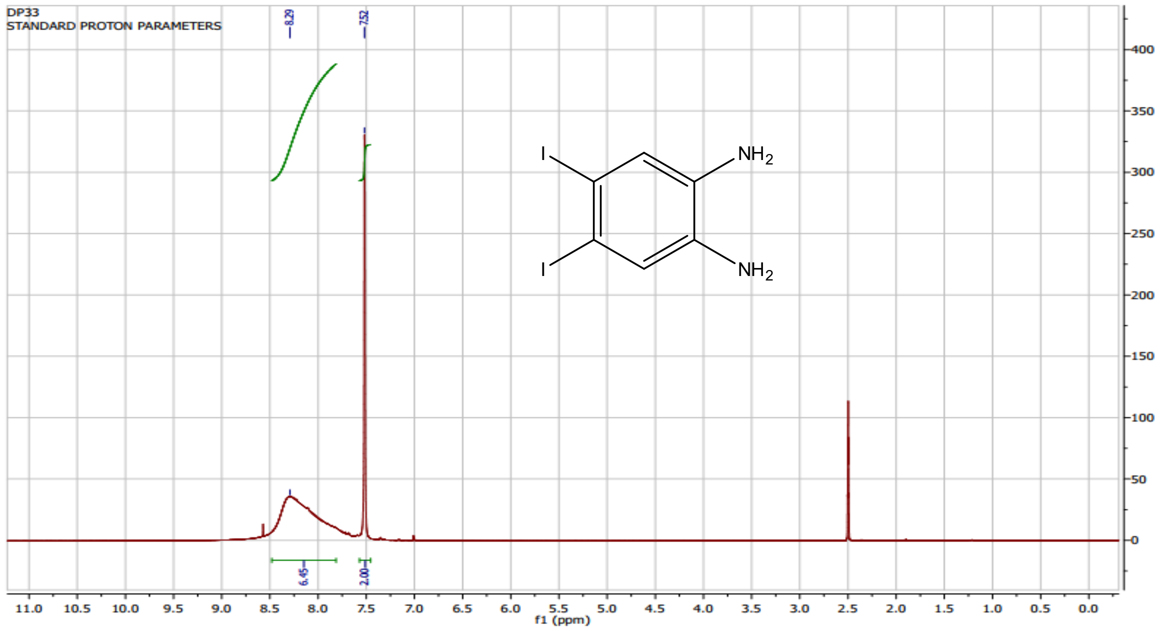

Figure S2. ^{1}H NMR spectrum of 1,2-diamino-4,5-diiodobenzene.

Synthesis of 2,1,3-benzothiadiazole derivatives (2a-e)

General procedure:

Benzene-1,2-diamine derivative (5 mmol) was dissolved in dry DCM (15 mL). Triethylamine (3 mL) was added, then thionyl chloride (15 mmol, 1.1 mL) dissolved in 3 mL of dry DCM was added dropwise at 0 °C. The reaction mixture was allowed to reach room temperature, then it was heated at 40 °C overnight. Afterwards, the reaction mixture was filtered. The filtrate was evaporated and the crude product was purified by column chromatography on silica gel using DCM as eluent.

2,1,3-benzothiadiazole (2a)

Yield = 66 %. **^{1}H NMR** (400 MHz, d-DMSO) δ 7.62-7.72 (2H, m, Ar*H*), 7.99-8.80 (2H, m, Ar*H*); **^{13}C NMR** (100 MHz, d-DMSO) δ 121.24, 129.83, 154.19; **HRMS** calcd. for $C_6H_5N_2S$ [M+H]$^+$: 137.01680, found: 137.01673.

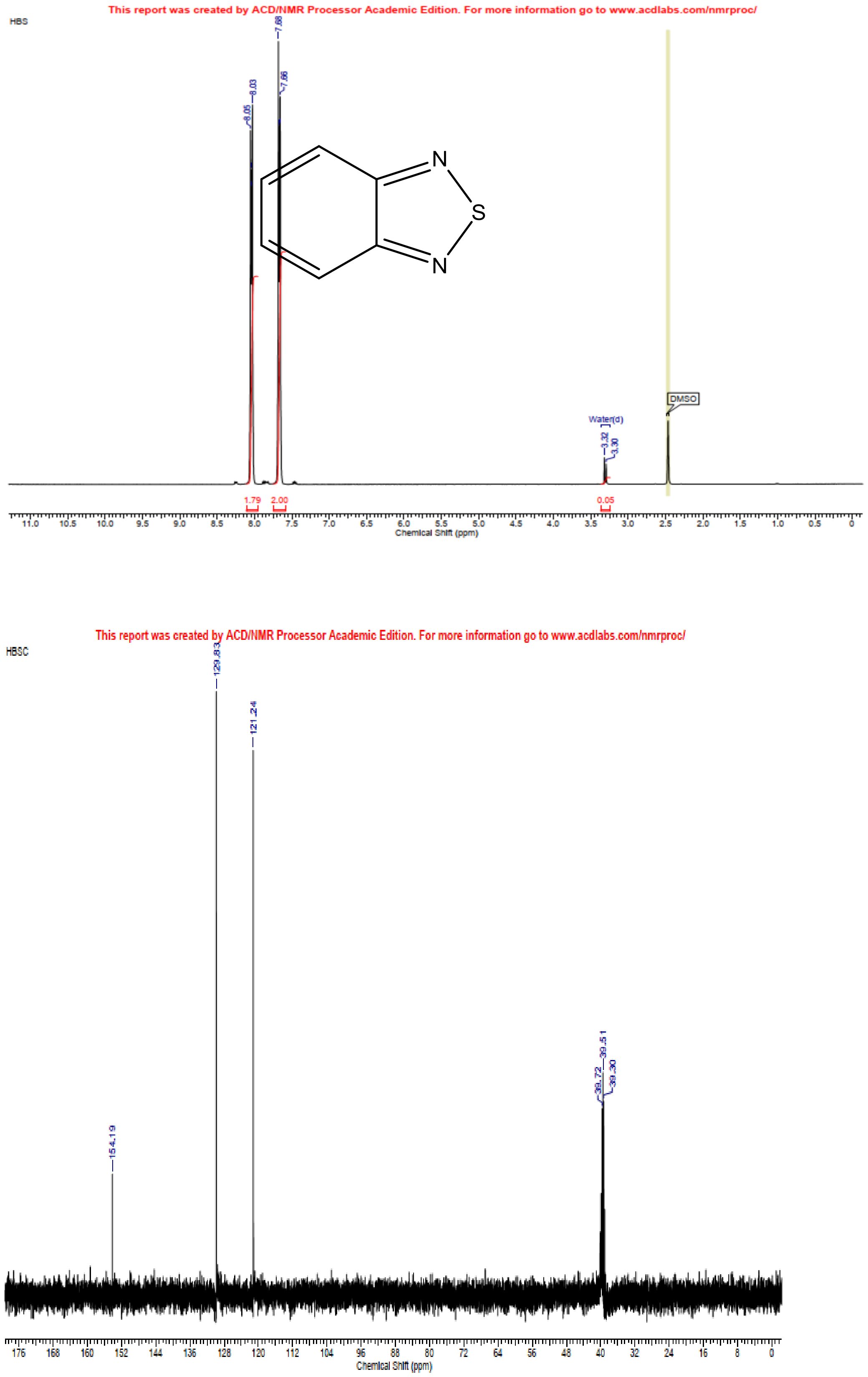

Figure S3. ^{1}H NMR spectrum of 2,1,3-benzothiadiazole (above) and ^{13}C NMR spectrum (below).

5,6-difluoro-2,1,3-benzothiadiazole (2b)

Yield = 66 %. ^{1}**H NMR** (500 MHz, d-DMSO) δ 8.18-8.27 (2H, m, Ar*H*); ^{13}**C NMR** (125 MHz, d-DMSO) δ 106.37, 106.54, 150.46, 1651.77, 151.94, 153.81, 153.97; **HRMS** calcd. for $C_6H_3F_2N_2S$ $[M+H]^+$: 172.99795, found: 172.99803.

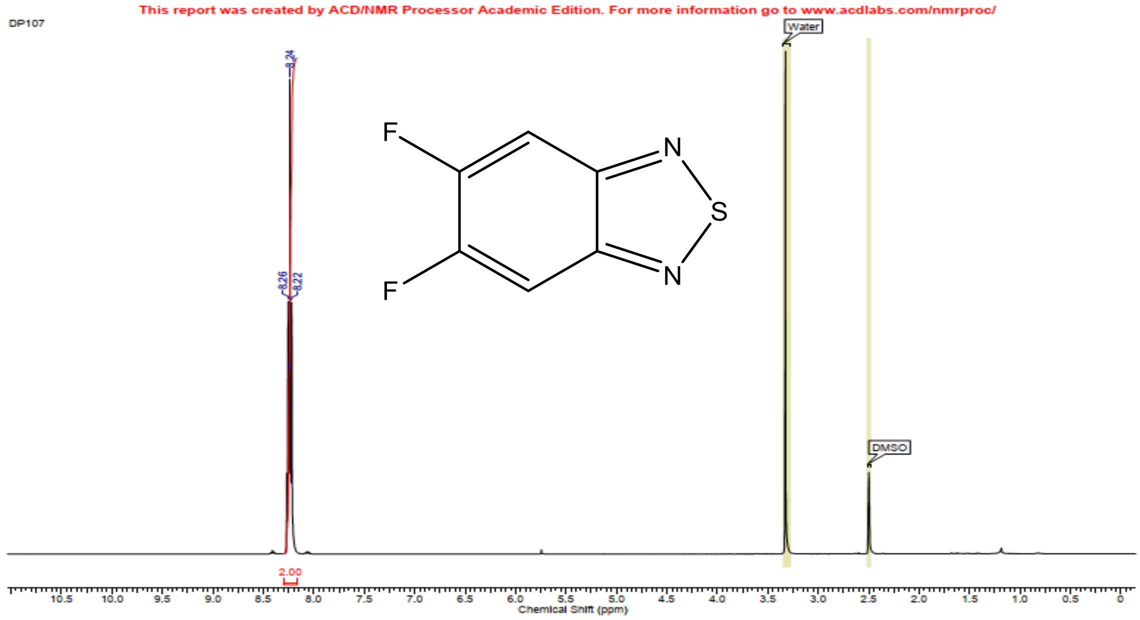

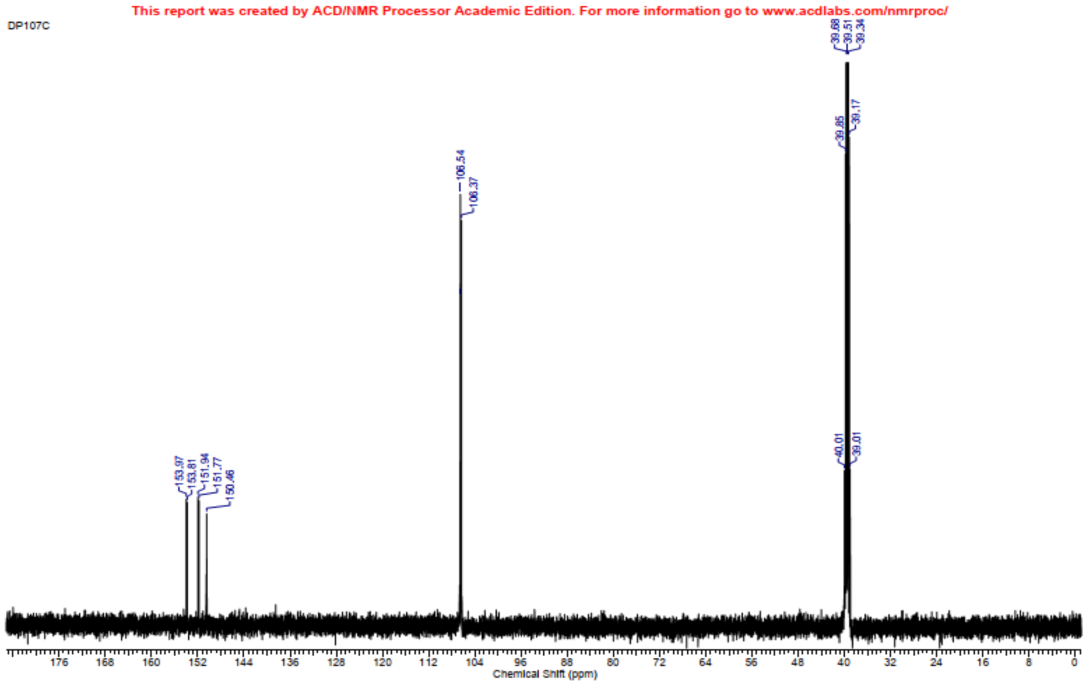

Figure S4. ^{1}H NMR spectrum of 5,6-difluoro-2,1,3-benzothiadiazole (above) and ^{13}C NMR spectrum (below).

5,6-dichloro-2,1,3-benzothiadiazole (2c)

Yield = 75 %. ^{1}**H NMR** (500 MHz, d-DMSO) δ 8.53 (2H, s, Ar*H*); ^{13}**C NMR** (125 MHz, d-DMSO) δ 121.75, 133.54, 152.61; **HRMS** calcd. for $C_6H_3Cl_2N_2S$ $[M+H]^+$: 204.93885, found: 204.93872.

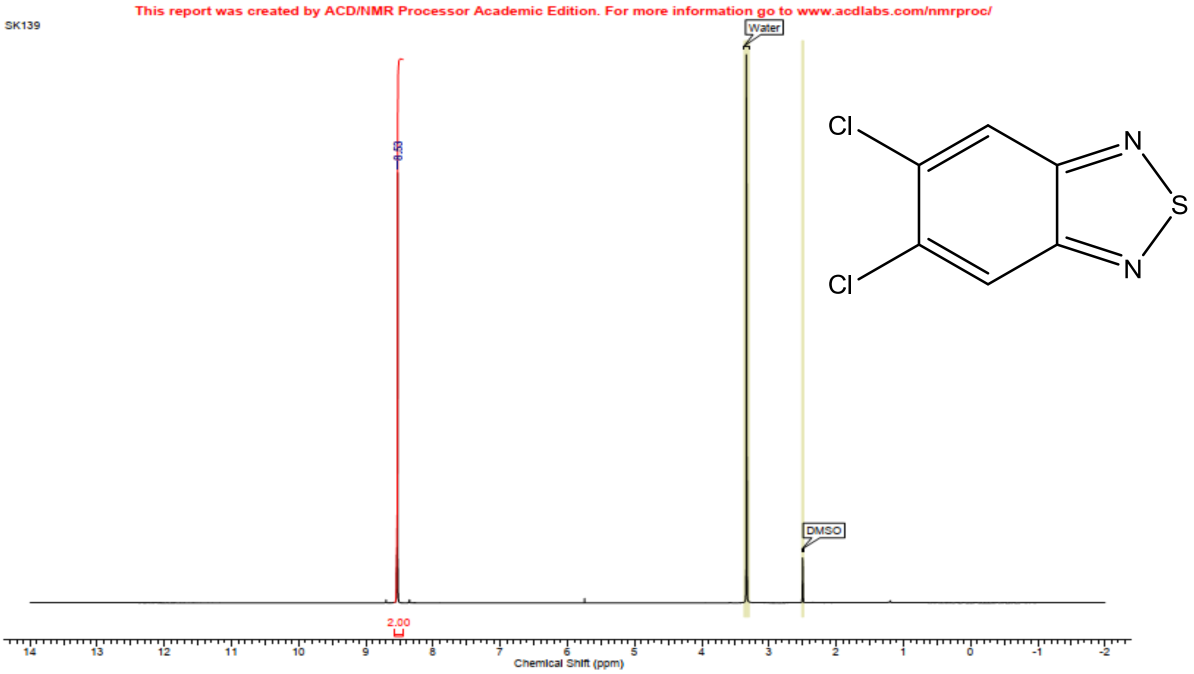

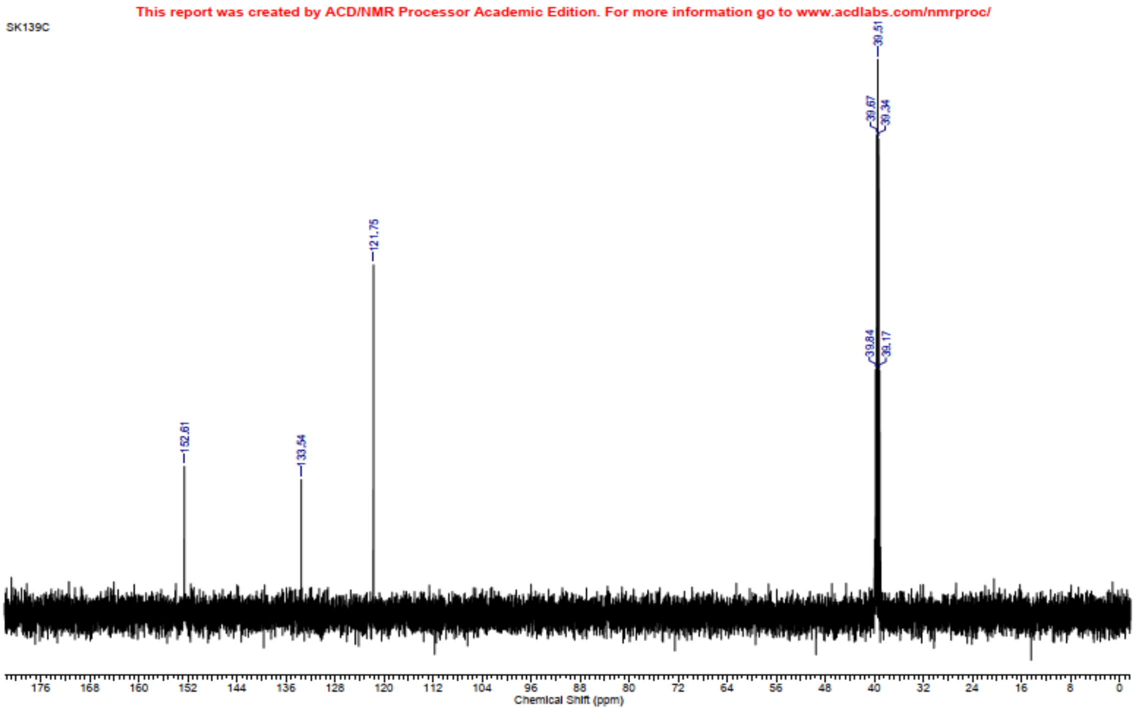

Figure S5. ^{1}H NMR spectrum of 5,6-dichloro-2,1,3-benzothiadiazole (above) and ^{13}C NMR spectrum (below).

5,6-dibromo-2,1,3-benzothiadiazole (2d)

Yield = 78 %. **^{1}H NMR** (500 MHz, d-DMSO) δ 8.67 (2H, s, Ar*H*); **^{13}C NMR** (125 MHz, d-DMSO) δ 124.95, 126.26, 153.32; **HRMS** calcd. for $C_6H_3Br_2N_2S$ $[M+H]^+$: 292.83782, found: 292.83741.

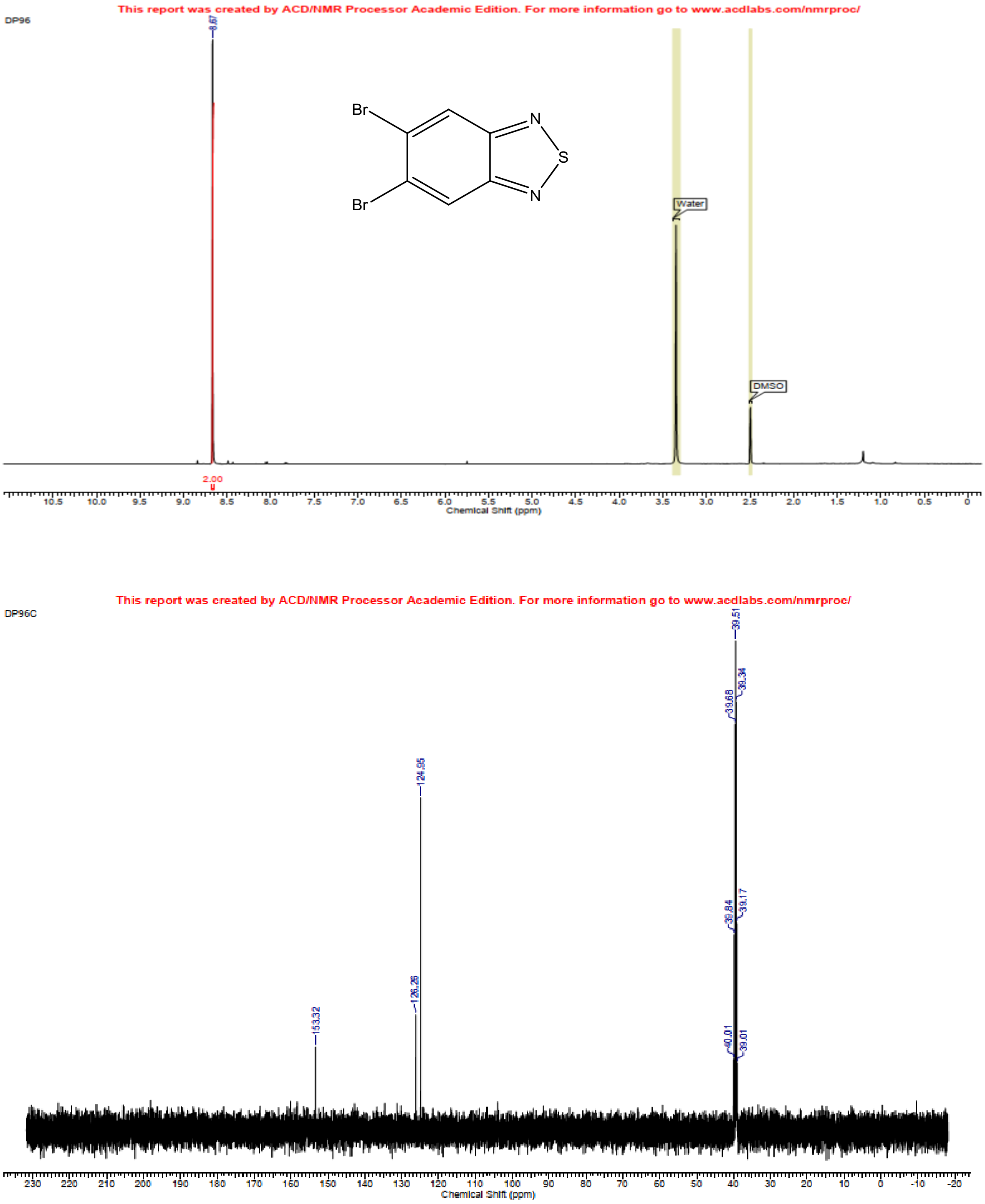

Figure S6. ^{1}H NMR spectrum of 5,6-dibromo-2,1,3-benzothiadiazole (above) and ^{13}C NMR spectrum (below).

5,6-diiodo-2,1,3-benzothiadiazole (2e)

Yield = 47 %. ^{1}**H NMR** (500 MHz, d-DMSO) δ 8.79 (2H, s, Ar*H*); ^{13}**C NMR** (125 MHz, d-DMSO) δ 111.39, 130.38, 154.22; **HRMS** calcd. for $C_6H_3I_2N_2S$ $[M+H]^+$: 388.81008, found: 388.30973.

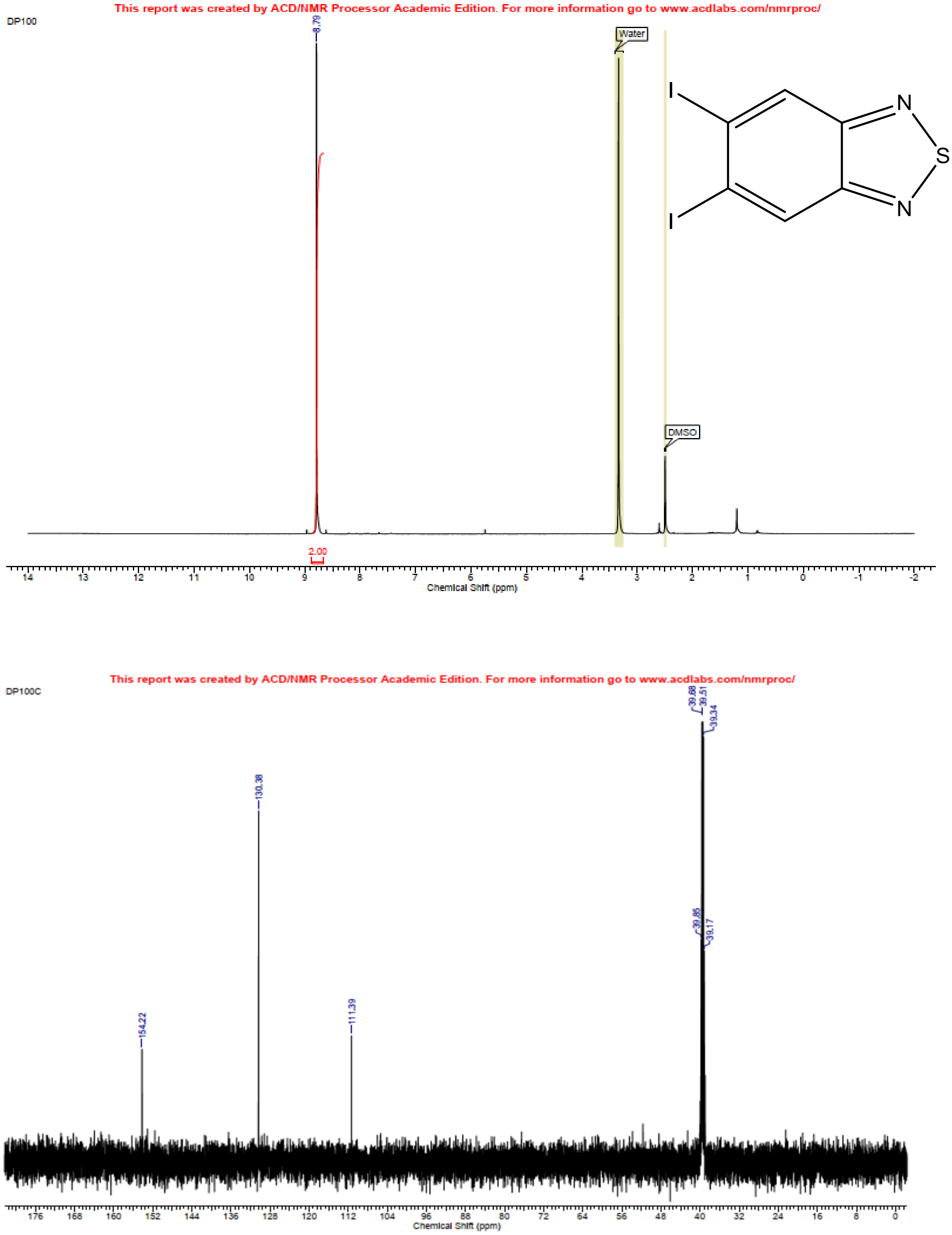

Figure S7. ^{1}H NMR spectrum of 5,6-diiodo-2,1,3-benzothiadiazole (above) and ^{13}C NMR spectrum (below).

Synthesis of 2-trifluoromethylo-1*H*-benzimidazole derivatives (3a-e)

General procedure:

Benzene-1,2-diamine derivative (2 mmol) was dissolved in trifluoroacetic acid (2 mL), a catalytic amount of concentrated HCl was added. The reaction mixture was heated at reflux overnight. The reaction was quenched by addition of 50 mL of concentrated $NaHCO_3$ solution, afterwards, it was extracted by ethyl acetate (3x30 mL). Combined organic layers were dried by $MgSO_4$, then solvent was evaporated. The crude product was purified by column chromatography on silica gel using 85:15 (hexane:AcOEt) as eluent (compounds **3d** and **3e**) or by recrystallization from hexane (compounds **3a**, **3b** and **3c**).

2-trifluoromethylo-1*H*-benzimidazole (3a)

Yield = 73 %. [1]**H NMR** (500 MHz, d-DMSO) δ 7.34-7.42(2H, m, Ar*H*), 7.68-7.77(2H, m, Ar*H*); [13]**C NMR** (125 MHz, d-DMSO) δ 115.88, 118.02, 120.17, 122.32, 124.09, 139.90, 140.22; **HRMS** calcd. for $C_8H_6F_3N_2$ $[M+H]^+$: 187.04776, found: 187.04773.

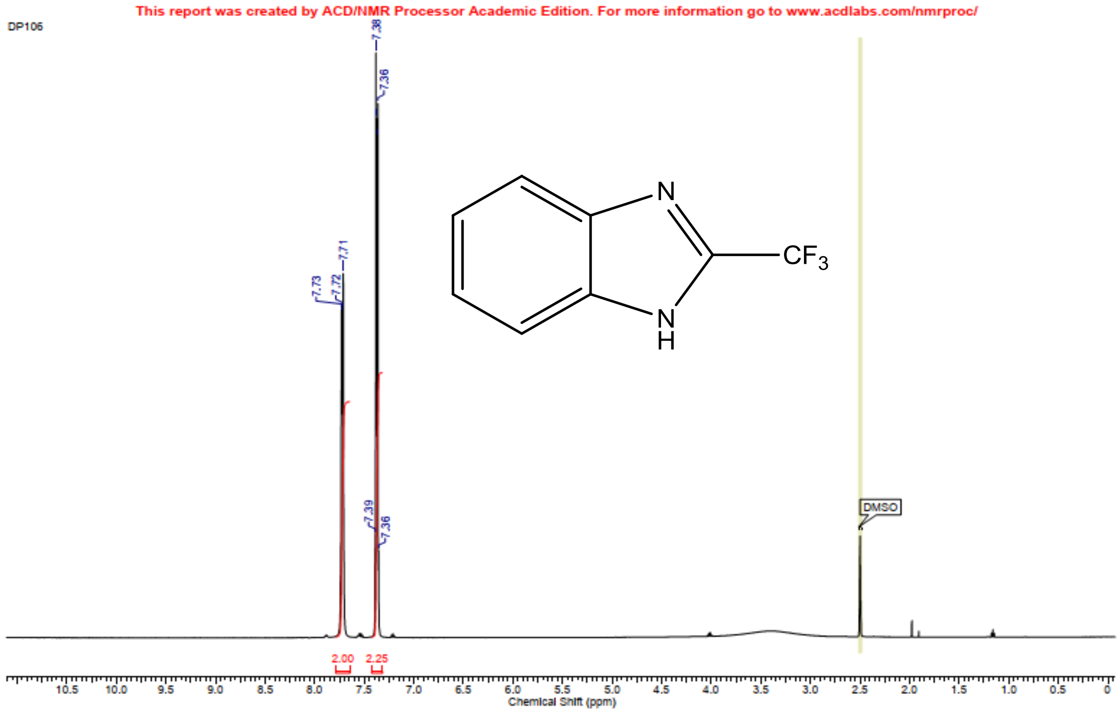

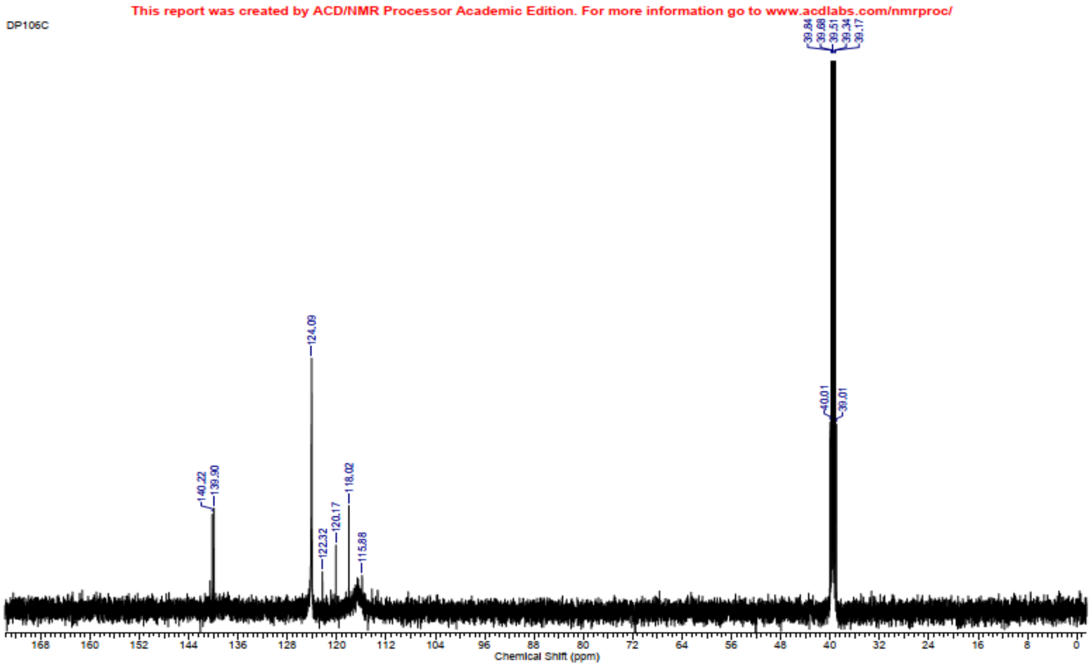

Figure S8. ^{1}H NMR spectrum of 2-trifluoromethylo-1*H*-benzimidazole (above) and ^{13}C NMR spectrum (below).

5,6-difluoro-2-trifluoromethylo-1*H*-benzimidazole (3b)

Yield = 67 %. ^{1}**H NMR** (500 MHz, d-DMSO) δ 7.71-7.85 (2H, m, Ar*H*); **HRMS** calcd. for $C_8H_4F_5N_2$ $[M+H]^+$: 223.02892, found: 223.02882.

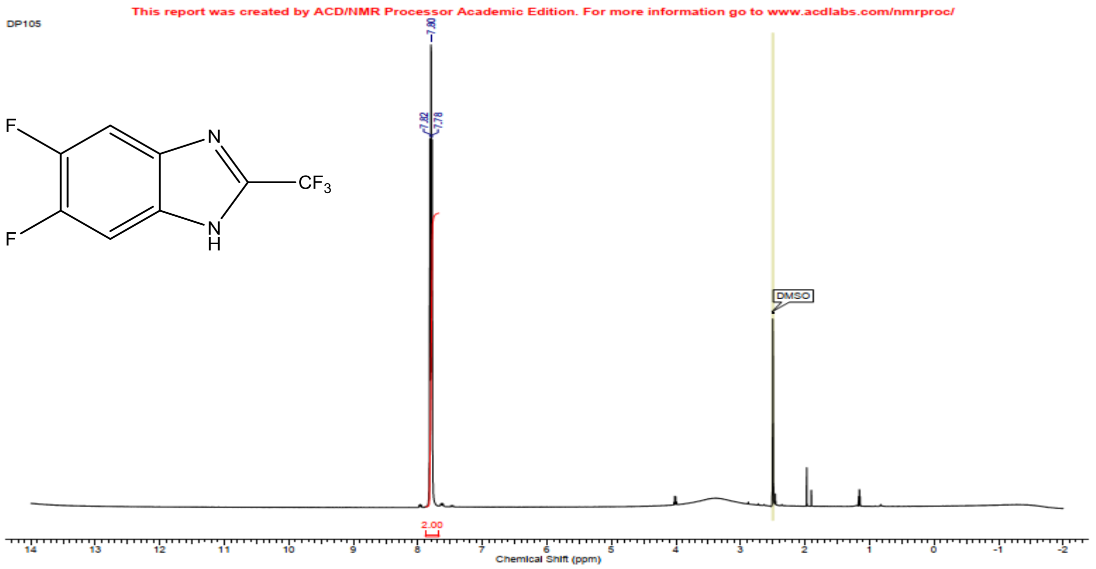

Figure S9. ^{1}H NMR spectrum of 5,6-difluoro-2-trifluoromethylo-1*H*-benzimidazole.

5,6-dichlorlo-2-trifluoromethylo-1*H*-benzimidazole (3c)

Yield = 85 %. ^{1}**H NMR** (500 MHz, d-DMSO) δ 8.00 (2H, s, Ar*H*); **HRMS** calcd. for $C_8H_4F_3Cl_2N_2$ $[M+H]^+$: 254.96981, found: 254.96976.

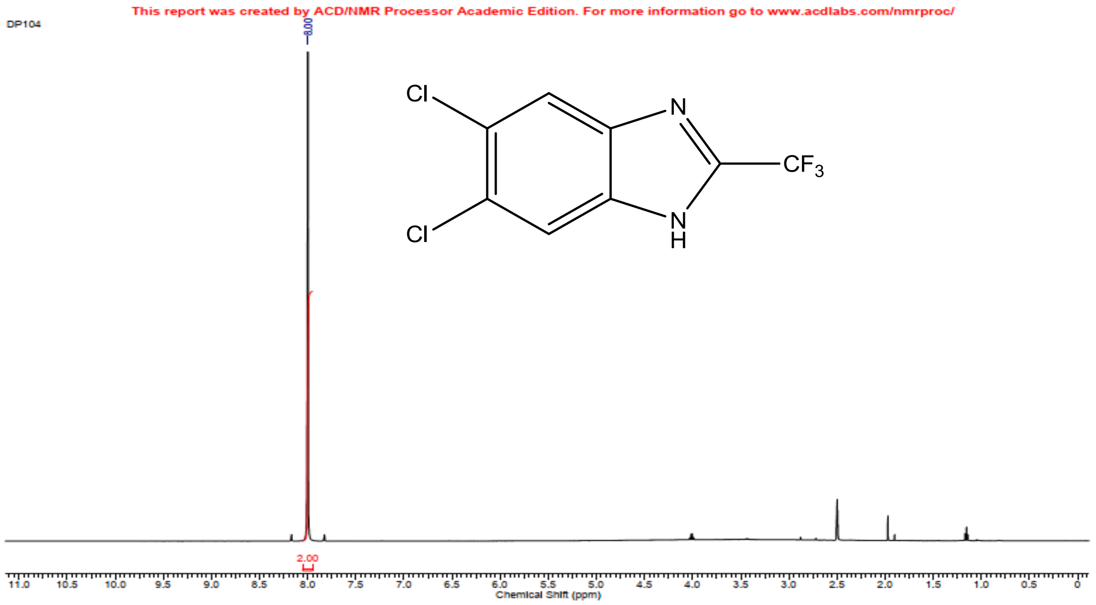

Figure S10. ^{1}H NMR spectrum of 5,6-dichloro-2-trifluoromethylo-1*H*-benzimidazole.

5,6-dibromo-2-trifluoromethylo-1*H*-benzimidazole (3d)

Yield = 40 %. **^{1}H NMR** (500 MHz, d-DMSO) δ 8.16 (2H, s, Ar*H*); **^{13}C NMR** (125 MHz, d-DMSO) δ 115.88,; **HRMS** calcd. for $C_8H_4F_3Br_2N_2$ $[M+H]^+$: 342.86878, found: 342.86896.

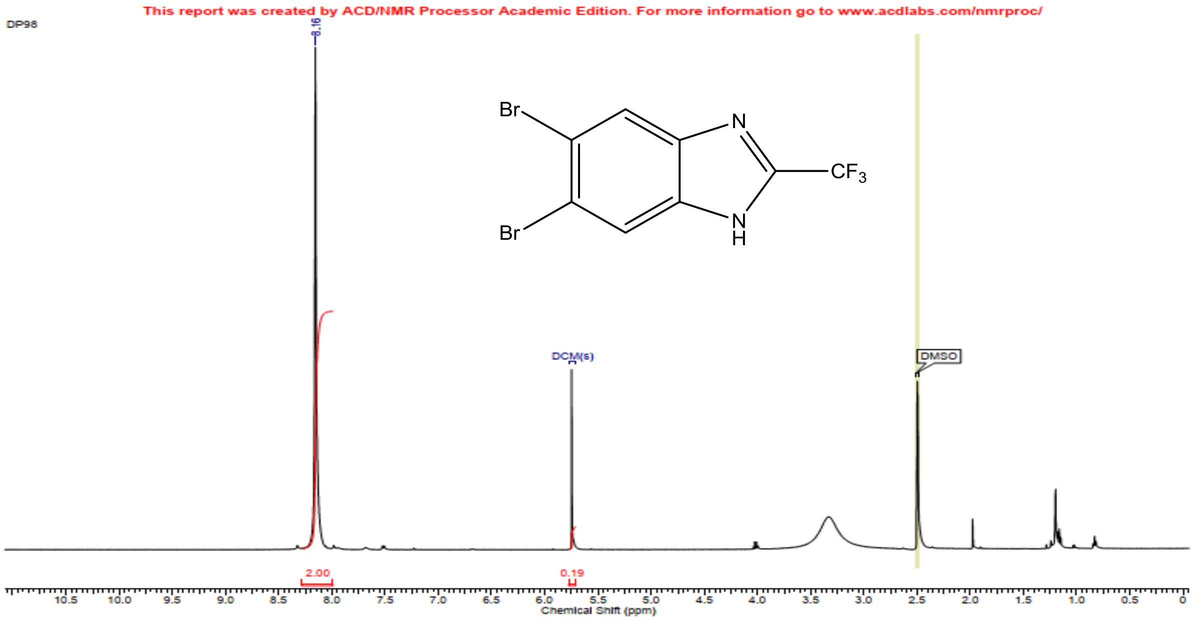

Figure S11. ^{1}H NMR spectrum of 5,6-dibromo-2-trifluoromethylo-1*H*-benzimidazole.

5,6-diiodo-2-trifluoromethylo-1*H*-benzimidazole (3e)

Yield = 33 %. **^{1}H NMR** (500 MHz, d-DMSO) δ 8.32 (2H, m, Ar*H*); **HRMS** calcd. for $C_8H_4F_3I_2N_2$ $[M+H]^+$: 438.84105, found: 438.84123.

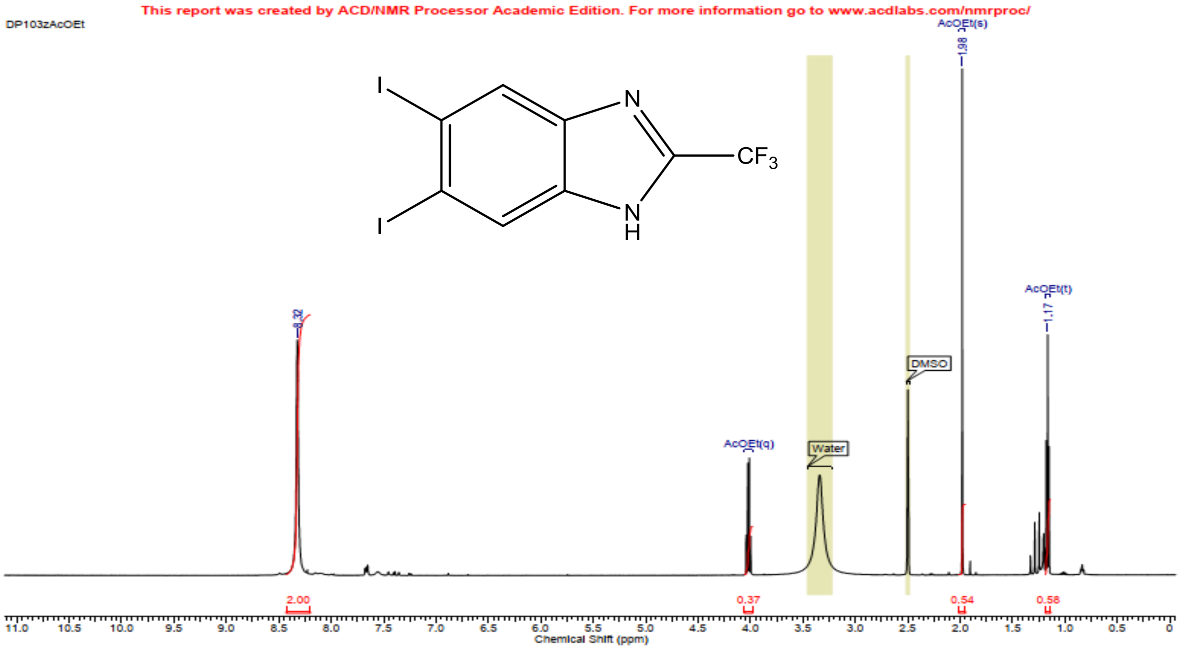

Figure S12. ^{1}H NMR spectrum of 5,6-diiodo-2-trifluoromethylo-1*H*-benzimidazole.

Synthesis of 2-hydroxymethylo-1*H*-benzimidazole derivatives (4a,c-e)

General procedure:

Benzene-1,2-diamine derivative (5 mmol) and hydroxyacetic acid (15 mmol, 1,14 g) were dissolved in water (1.5 mL), concentrated HCl (0.5 mL) was added. The reaction mixture was heated at reflux overnight. The reaction was cooled to RT, then 20% NaOH solution was added until pH = 13. The participate was filtered and washed several times with water. Compound **4e** was additionally purified by recrystallization from MeOH.

2-hydroxymethylo-1*H*-benzimidazole (4a)

Yield = 85 %. ^{1}**H NMR** (500 MHz, d-DMSO) δ 4.70 (2H, s, C*H*$_2$), 5.69 (1H, s br, O*H*), 7.11-7.15 (2H, m, Ar*H*), 7.49 (2H, s br, Ar*H*), 12.30 (1H, s br, N*H*); ^{13}**C NMR** (125 MHz, d-DMSO) δ 57.74, 121.25, 155.02; **HRMS** calcd. for $C_8H_9N_2O$ [M+H]$^+$: 149.07094, found: 149.07089.

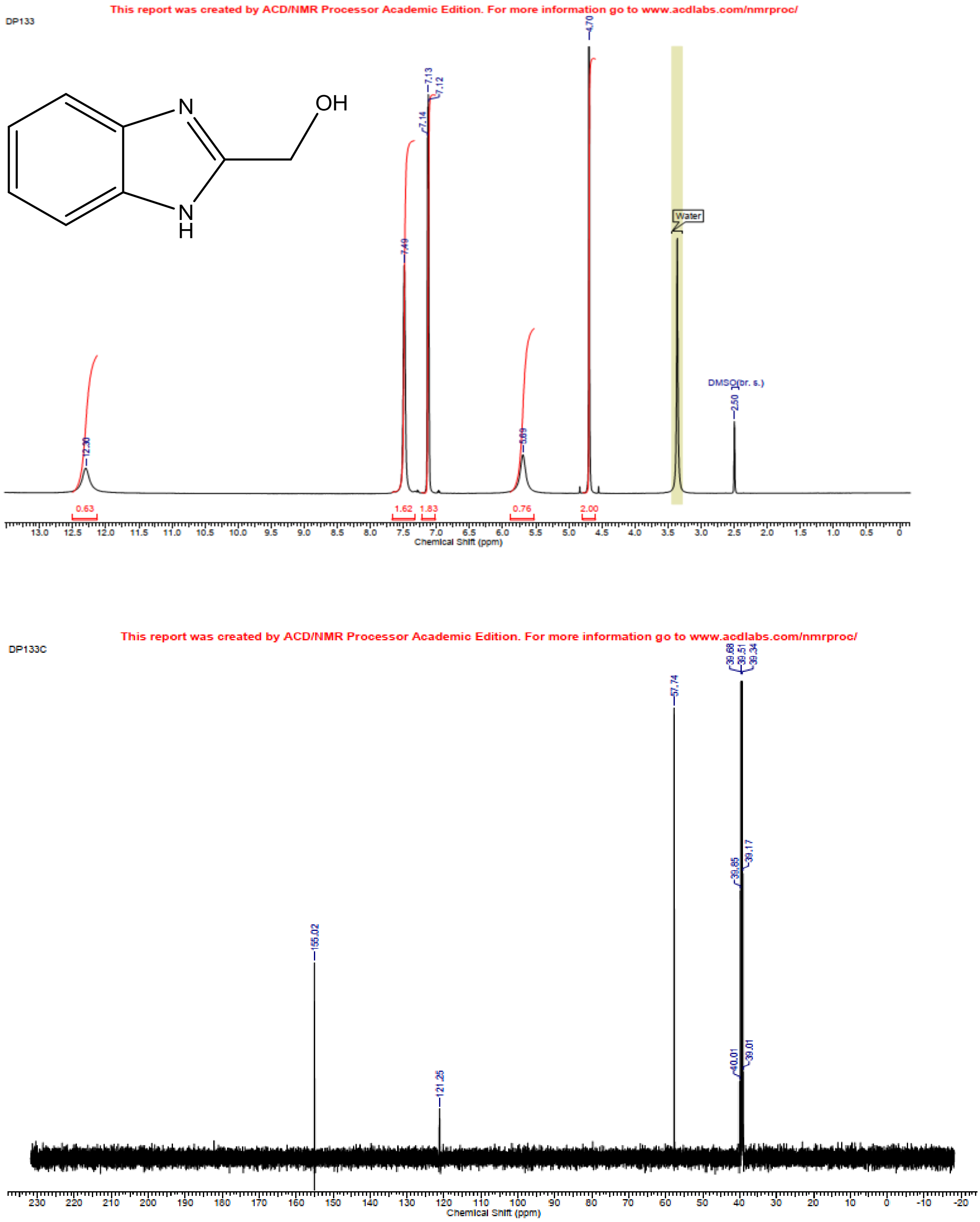

Figure S13. ^{1}H NMR spectrum of 2-hydroxymethylo-1*H*-benzimidazole (above) and ^{13}C NMR spectrum (below).

5,6-dichloro-2-hydroxymethylo-1*H*-benzimidazole (4c)

Yield = 84 %. ^{1}**H NMR** (500 MHz, d-DMSO) δ 4.70 (2H, s, CH_2), 5.81 (1H, s br, O*H*), 7.73 (2H, s br, Ar*H*), 12.04 (1H, s br, N*H*); ^{13}**C NMR** (125 MHz, d-DMSO) δ 57.57, 123.71, 158.10; **HRMS** calcd. for $C_8H_7Cl_2N_2O$ $[M+H]^+$: 216.99299, found: 216.99308.

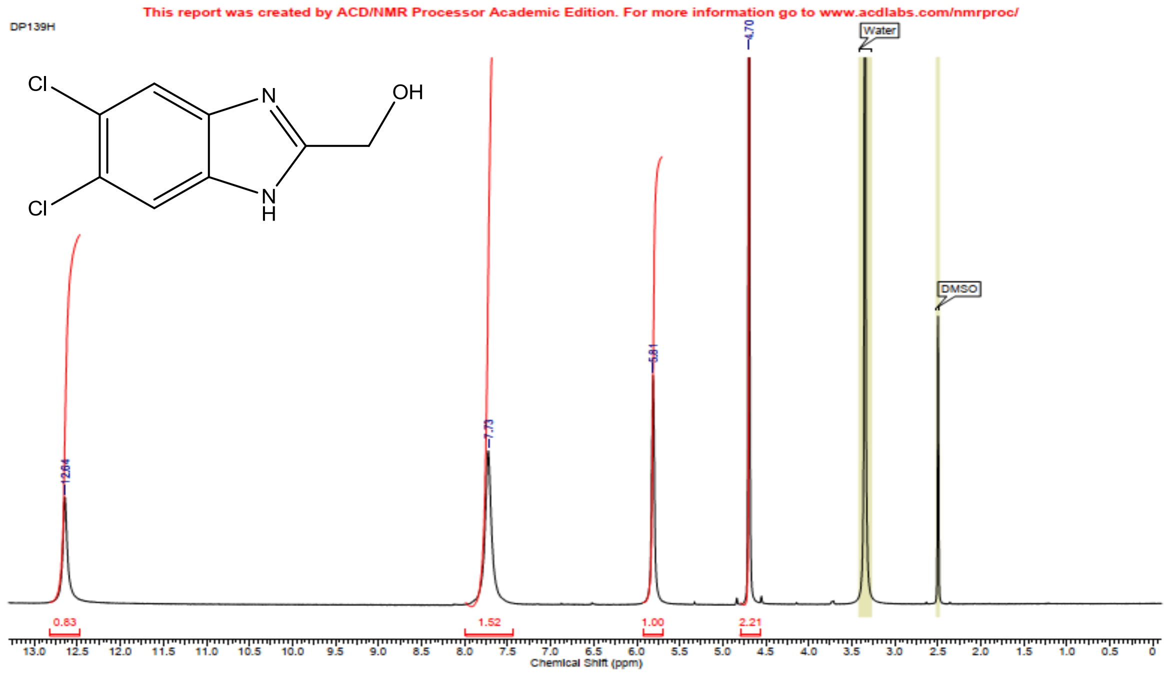

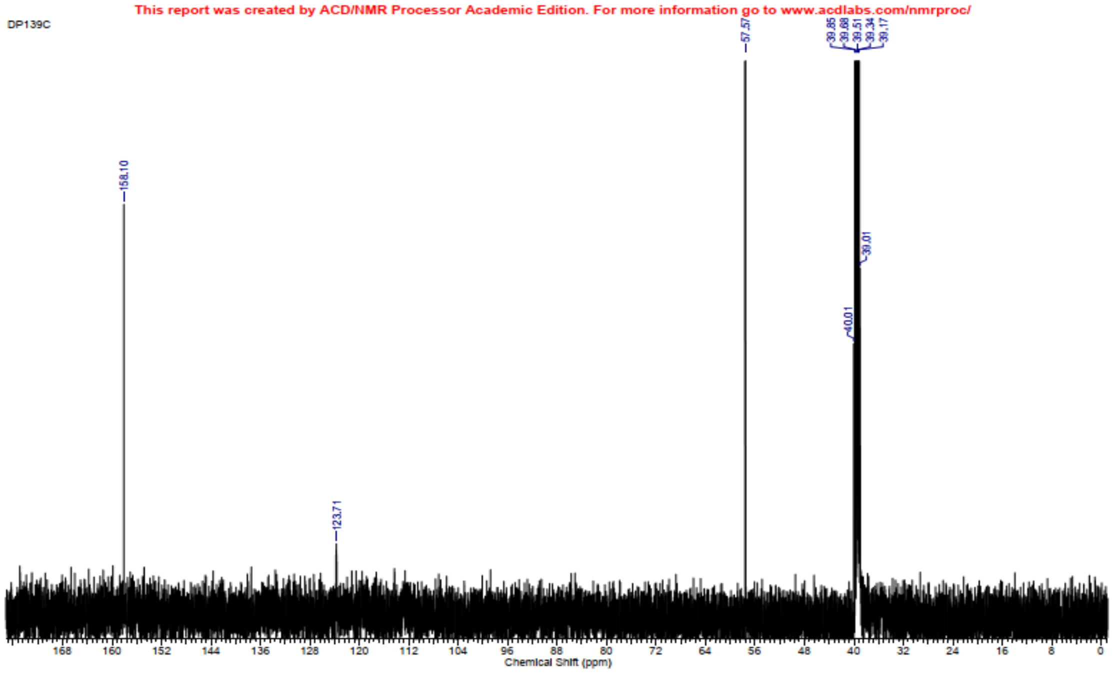

Figure S14. ^{1}H NMR spectrum of 5,6-dichloro-2-hydroxymethylo-1*H*-benzimidazole (above) and ^{13}C NMR spectrum (below).

5,6-dibromo-2-hydroxymethylo-1*H*-benzimidazole (4d)

Yield = 81 %. [1]**H NMR** (500 MHz, d-DMSO) δ 4.70 (2H, s, CH_2), 5.83 (1H, s br, O*H*), 7.88 (2H, s br, Ar*H*); [13]**C NMR** (125 MHz, d-DMSO) δ 57.50, 115.47, 119.18, 157.91; **HRMS** calcd. for $C_8H_7Br_2N_2O$ $[M+H]^+$: 304.89196, found: 304.89222.

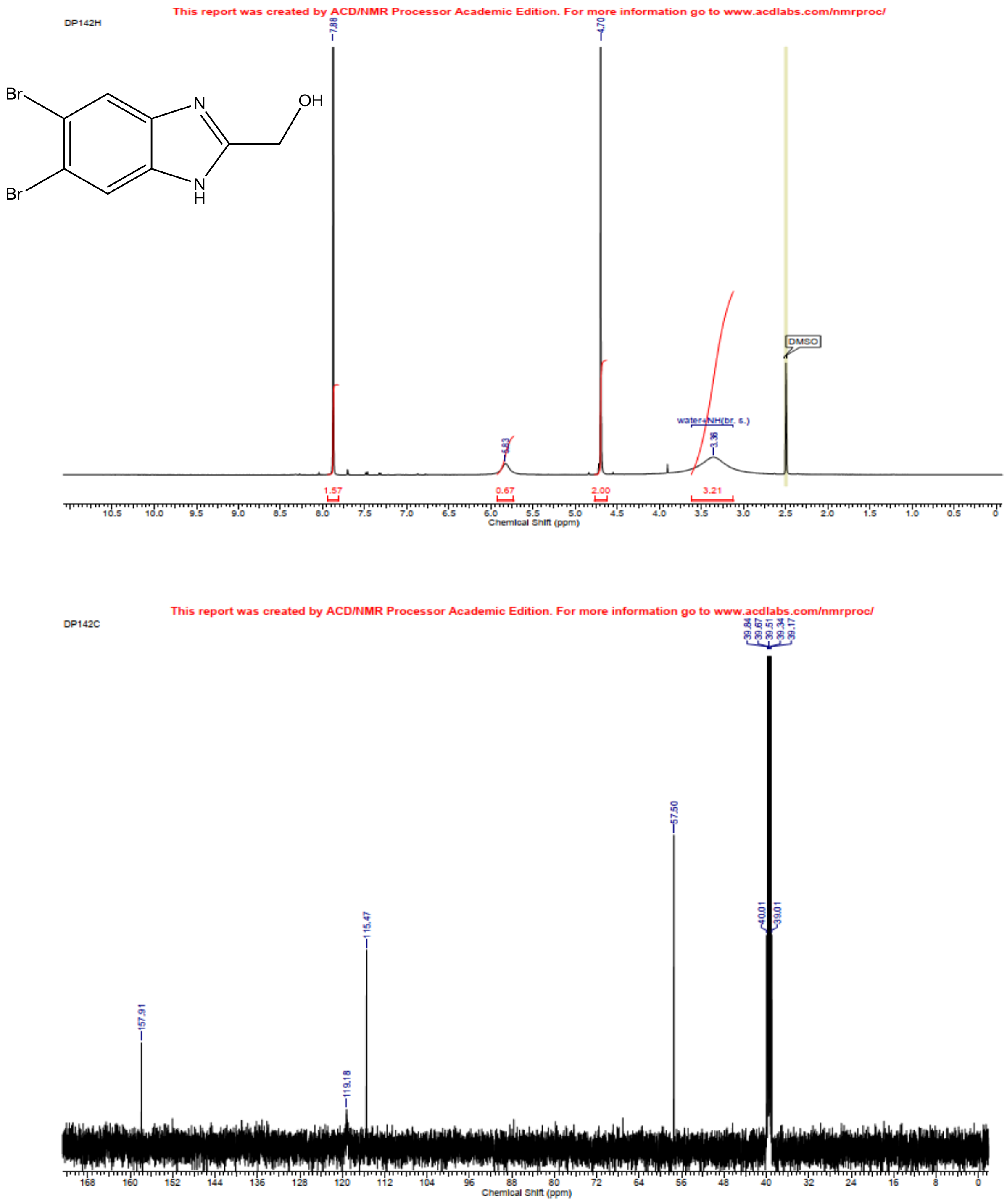

Figure S15. ^{1}H NMR spectrum of 5,6-dibromo-2-hydroxymethylo-1*H*-benzimidazole (above) and ^{13}C NMR spectrum (below).

5,6-diiodo-2-hydroxymethylo-1*H*-benzimidazole (4e)

Yield = 66 %. ^{1}**H NMR** (500 MHz, d-DMSO) δ 4.68 (2H, s, CH_2), 5.80 (1H, s br, O*H*), 8.07 (2H, s br, Ar*H*), 12.52 (1H, s br, N*H*); ^{13}**C NMR** (125 MHz, d-DMSO) δ 57.48, 98.13, 157.17; **HRMS** calcd. for $C_8H_7I_2N_2O$ $[M+H]^+$: 400.86423, found: 400.86446.

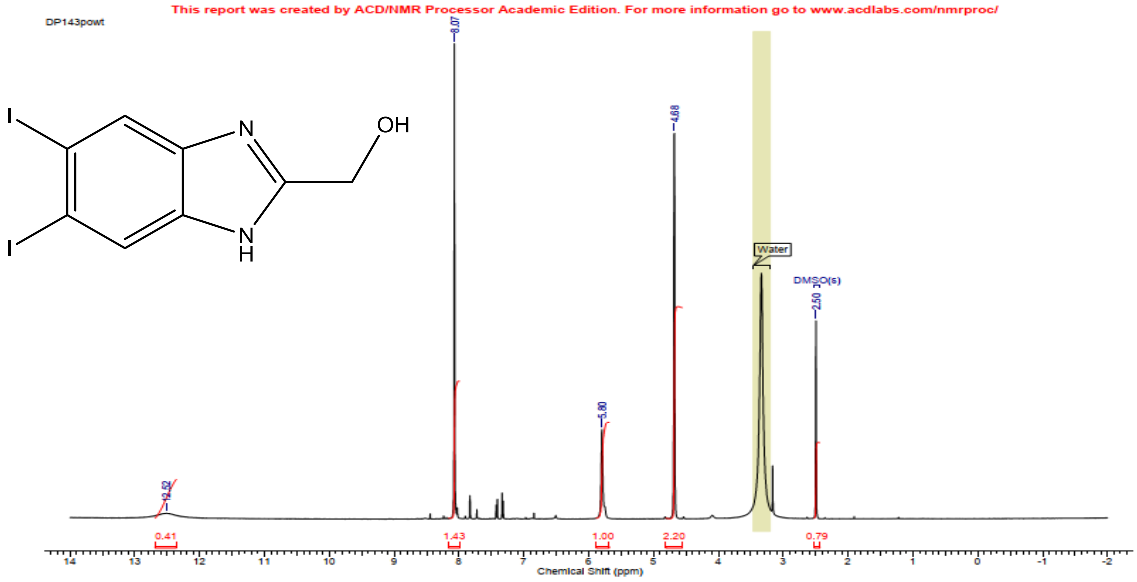

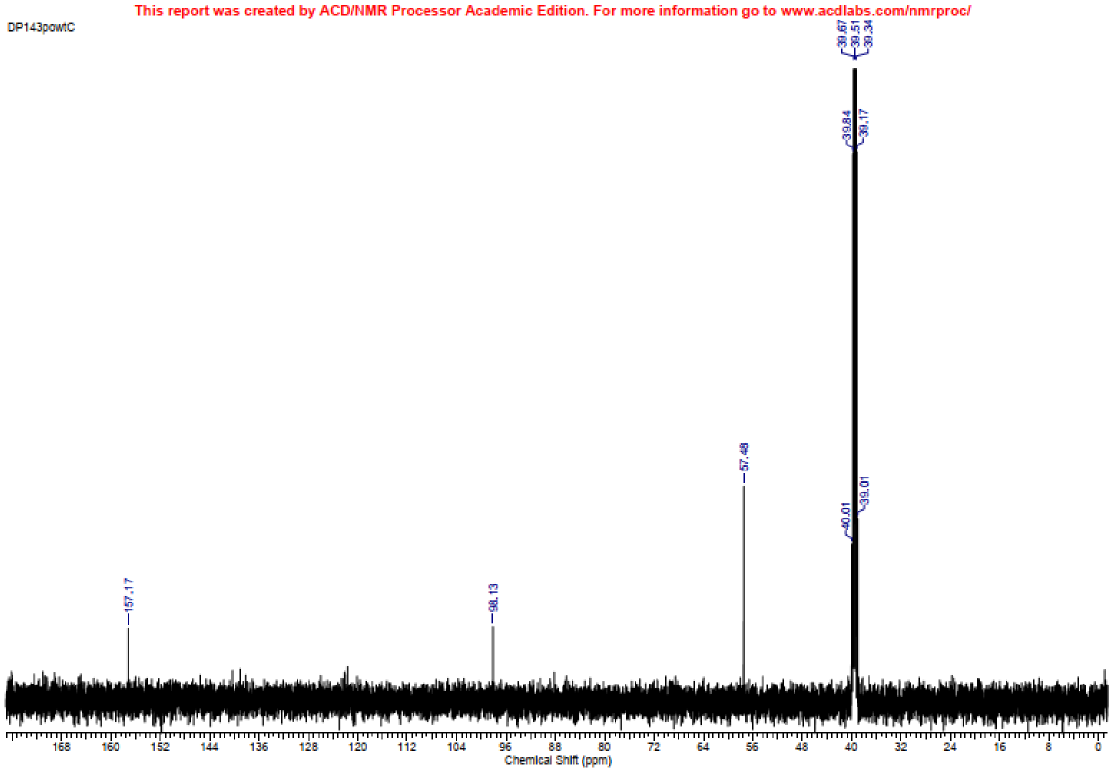

Figure S16. ^{1}H NMR spectrum of 5,6-diiodo-2-hydroxymethylo-1*H*-benzimidazole (above) and ^{13}C NMR spectrum (below).

Synthesis of 1*H*-benzo[d]imidazol-2(3*H*)-one derivatives (5a,c-e)

General procedure:

Benzene-1,2-diamine derivative (3 mmol) and *N*,*N*′-carbonyldiimidazole (CDI) (3 mmol, 486 mg) were mixed in DMF (4 mL) overnight at RT. The reaction was quenched by addition of water (20 mL). The participate was filtered and washed several times with water. Compound **5d** was additionally purified by recrystallization from ethyl acetate, then MeOH.

1*H*-benzo[d]imidazol-2(3*H*)-one (5a)

Yield = 45 %. ^{1}**H NMR** (500 MHz, d-DMSO) δ 6.91 (4H, s br, Ar*H*), 10.56 (2H, s br, N*H*); ^{13}**C NMR** (125 MHz, d-DMSO) δ 108.46, 120.40, 129.65, 155.25; **HRMS** calcd. for $C_7H_7N_2O$ $[M+H]^+$: 135.05529, found: 135.05526.

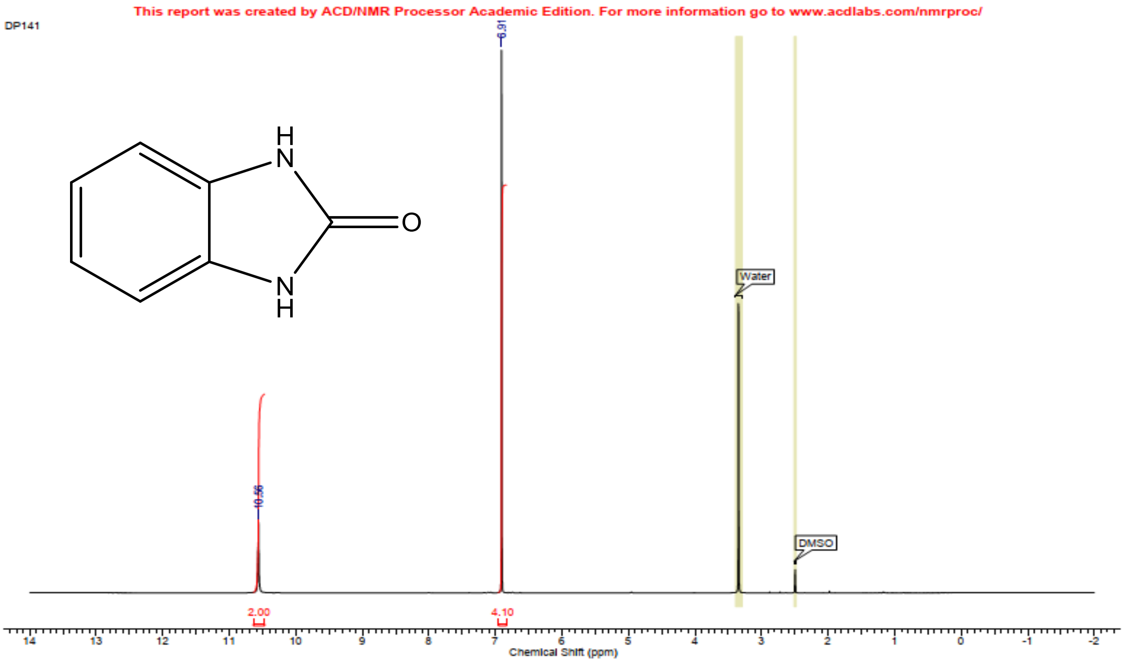

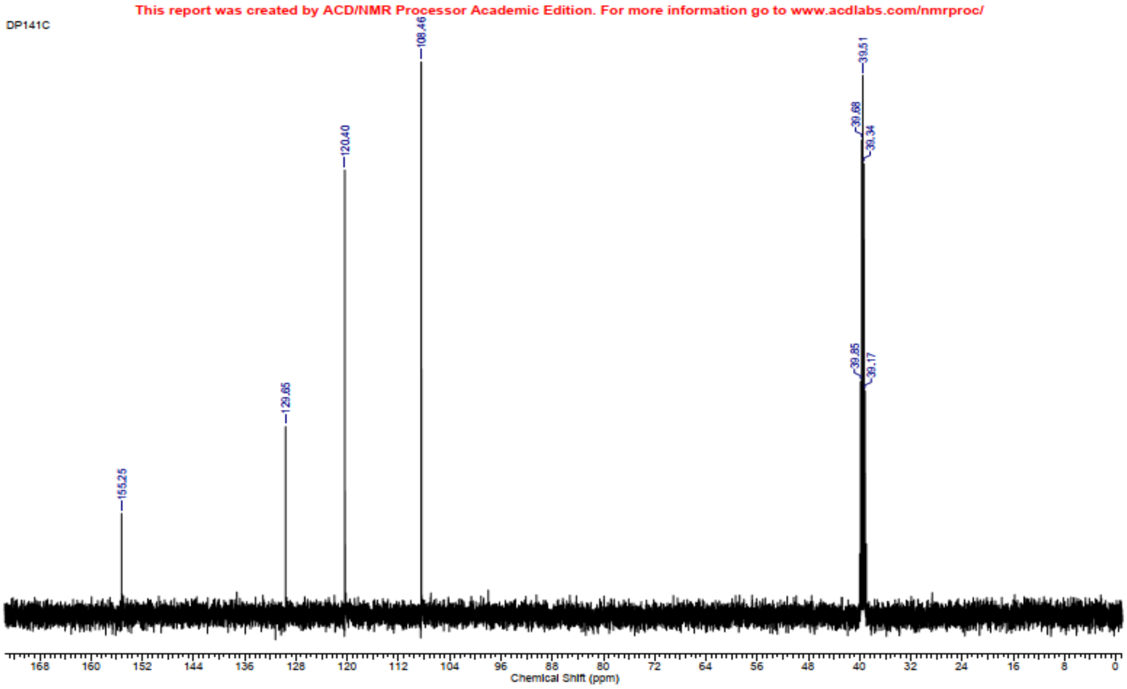

Figure S17. ^{1}H NMR spectrum of 1*H*-benzo[d]imidazol-2(3*H*)-one (above) and ^{13}C NMR spectrum (below).

5,6-dichloro-1*H*-benzo[d]imidazol-2(3*H*)-one (5c)

Yield = 67 %. ^{1}**H NMR** (500 MHz, d-DMSO) δ 7.08 (2H, s br, Ar*H*), 10.91 (2H, s br, N*H*); ^{13}**C NMR** (100 MHz, d-DMSO) δ 109.73, 122.35, 129.83, 155.18; **HRMS** calcd. for $C_7H_5Cl_2N_2O$ $[M+H]^+$: 202.97734, found: 202.97743.

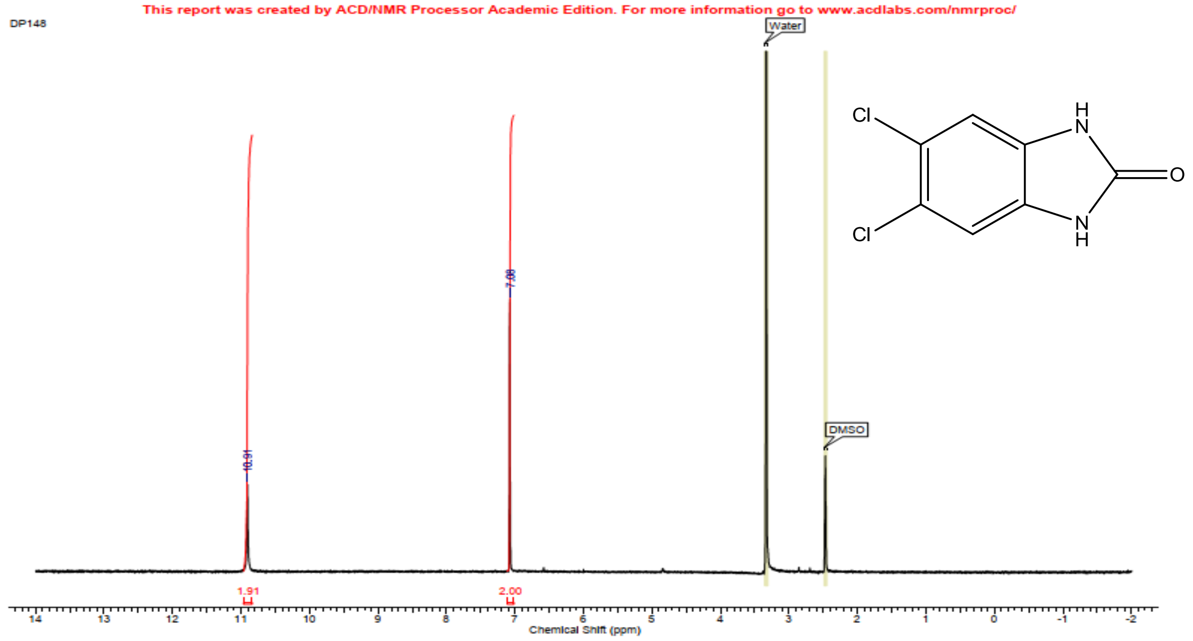

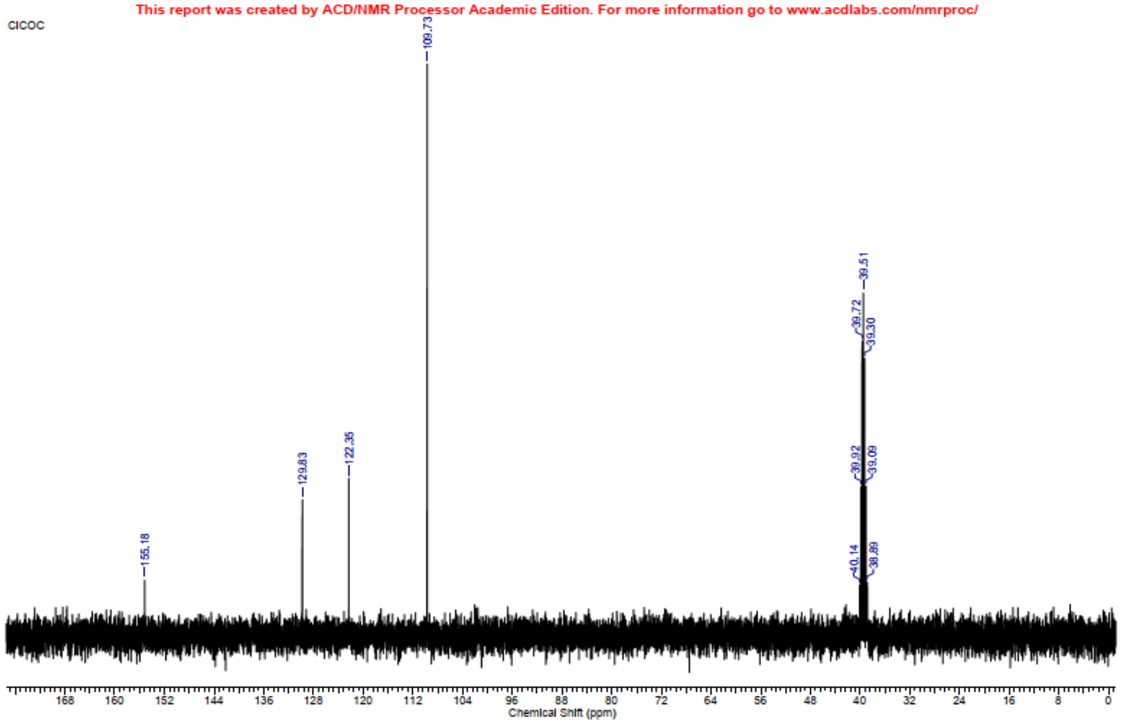

Figure S18. ^{1}H NMR spectrum of 5,6-dichloro-1*H*-benzo[d]imidazol-2(3*H*)-one (above) and ^{13}C NMR spectrum (below).

5,6-dibromo-1*H*-benzo[d]imidazol-2(3*H*)-one (5d)

Yield = 67 %. ^{1}**H NMR** (500 MHz, d-DMSO) δ 7.20 (2H, s br, Ar*H*), 10.90 (2H, s br, N*H*); ^{13}**C NMR** (100 MHz, d-DMSO) δ 112.66, 113.88, 130.63, 154.93; **HRMS** calcd. for $C_7H_5Br_2N_2O$ $[M+H]^+$: 290.87631, found: 290.87626.

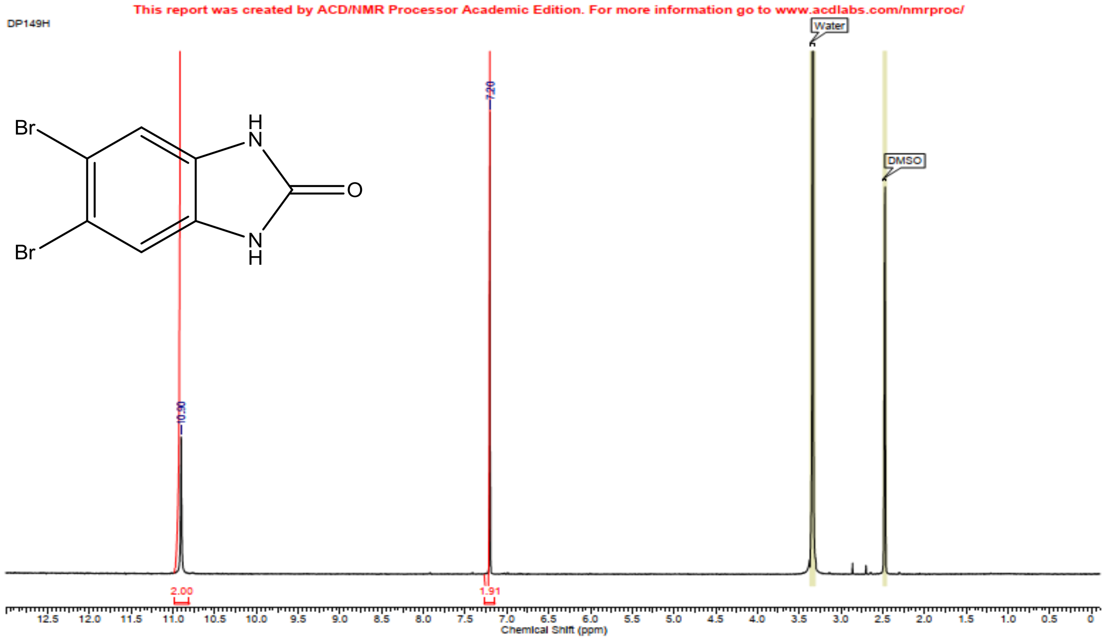

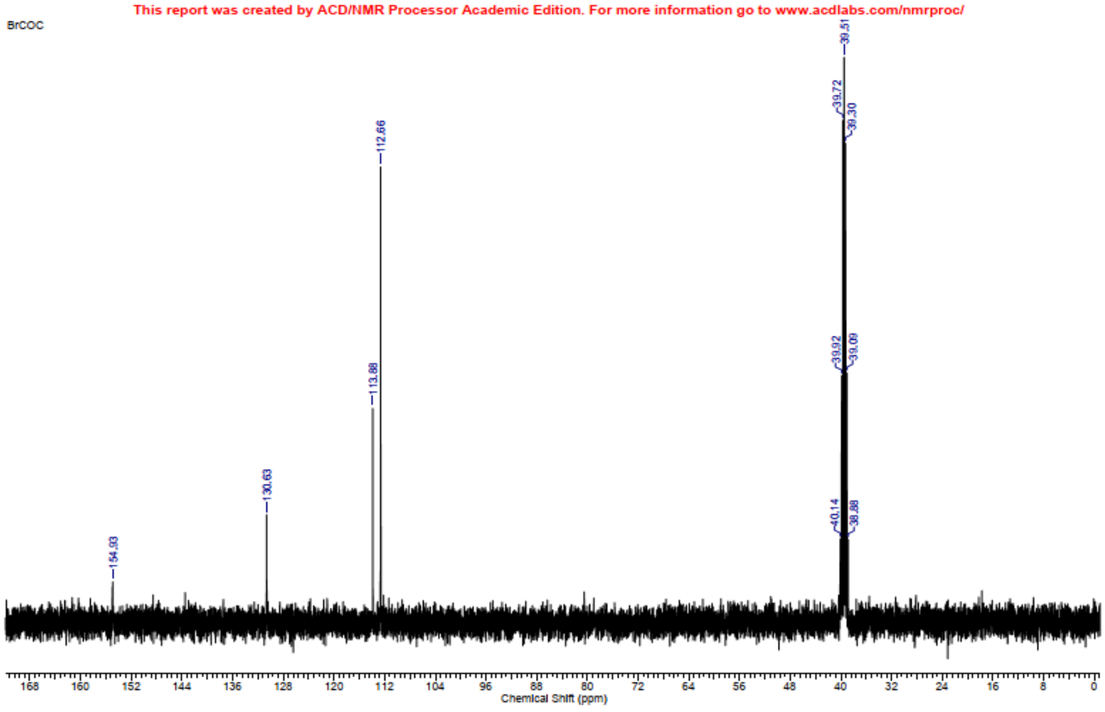

Figure S19. ^{1}H NMR spectrum of 5,6-dibromo-1*H*-benzo[d]imidazol-2(3*H*)-one (above) and ^{13}C NMR spectrum (below).

5,6-diiodo-1*H*-benzo[d]imidazol-2(3*H*)-one (5e)

Yield = 67 %. ^{1}**H NMR** (500 MHz, d-DMSO) δ 7.41 (2H, s br, Ar*H*), 10.80 (2H, s br, N*H*); ^{13}**C NMR** (100 MHz, d-DMSO) δ 96.40, 118.00, 131.46, 154.58; **HRMS** calcd. for $C_7H_5I_2N_2O$ $[M+H]^+$: 386.84858, found: 386.84861.

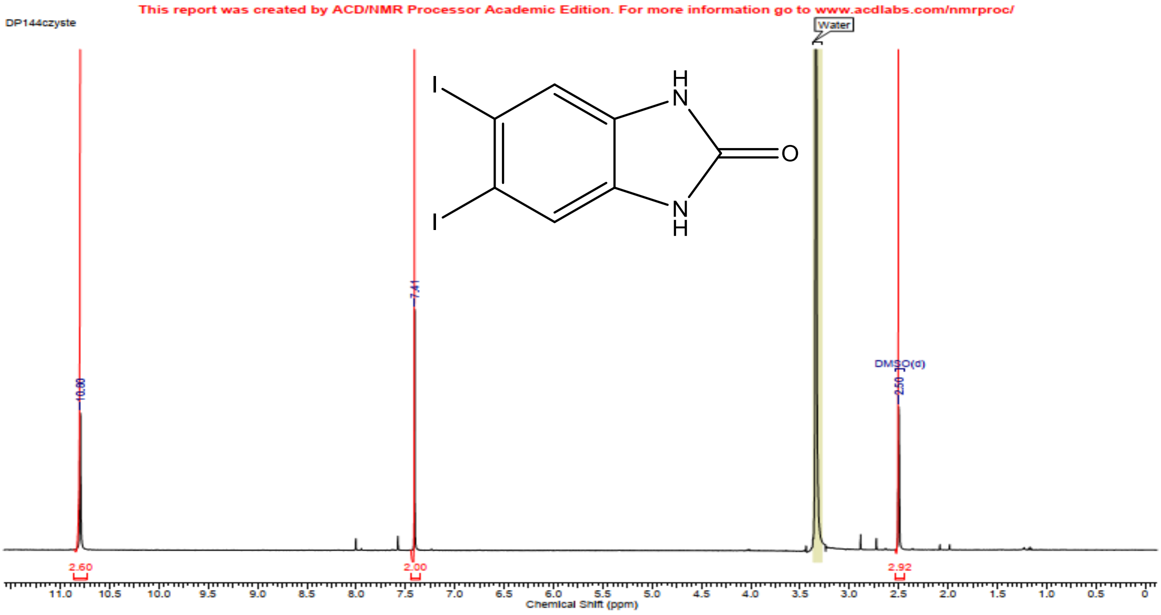

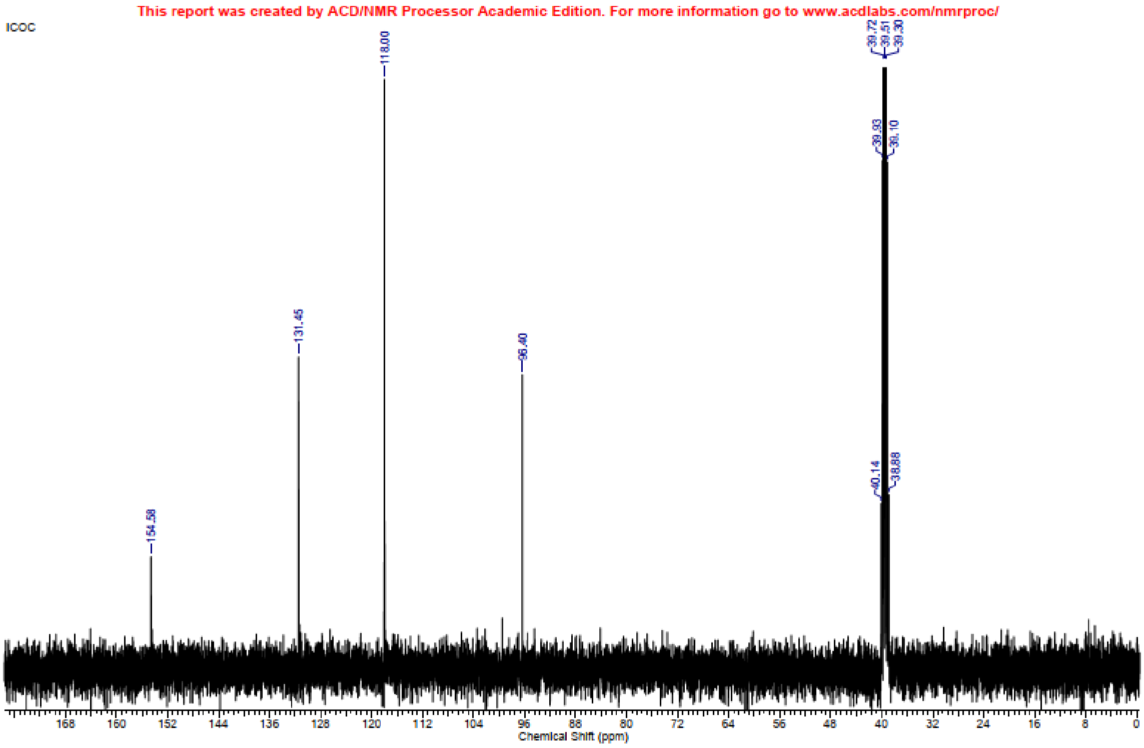

Figure S20. ^{1}H NMR spectrum of 5,6-diiodo-1*H*-benzo[d]imidazol-2(3*H*)-one (above) and ^{13}C NMR spectrum (below).

Synthesis of quinoxaline derivatives (6a,c-e)

General procedure:

Benzene-1,2-diamine derivative (3 mmol) and 40% glyoxal solution in water (9 mmol, 1.3 mL) were heated at reflux in EtOH (6 mL) overnight. The reaction was quenched by addition of water (50 mL). The participate was filtered and washed several times with water (compound **6a** was extracted with AcOEt (3x30 mL). Compounds **6c** and **6d** were purified by recrystallization from MeOH. Compounds **6a** and **6e** were purified by column chromatography on silica gel using 8:2 (hexane:AcOEt) as eluent.

quinoxaline (6a)

Yield = 70 %. ^{1}**H NMR** (500 MHz, d-DMSO) δ 7.84-7.92 (2H, m, Ar*H*), 8.08-8.16 (2H, m, Ar*H*), 8.96 (2H, s br, Ar*H*); ^{13}**C NMR** (125 MHz, d-DMSO) δ 129.14, 130.21, 142.23, 145.72; **HRMS** calcd. for $C_8H_7N_2$ $[M+H]^+$: 131.06037, found: 131.06042.

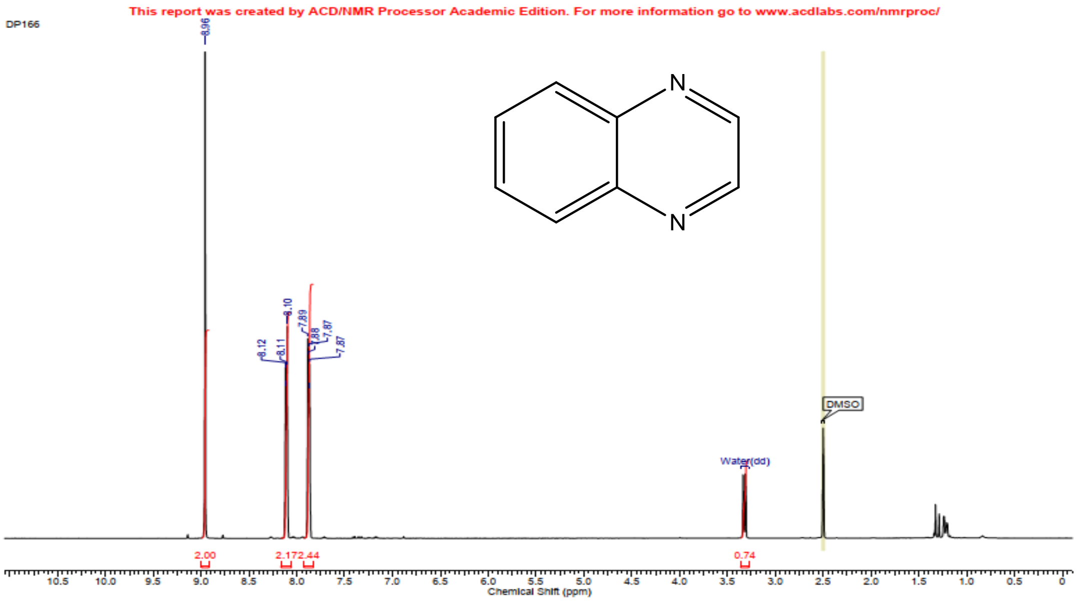

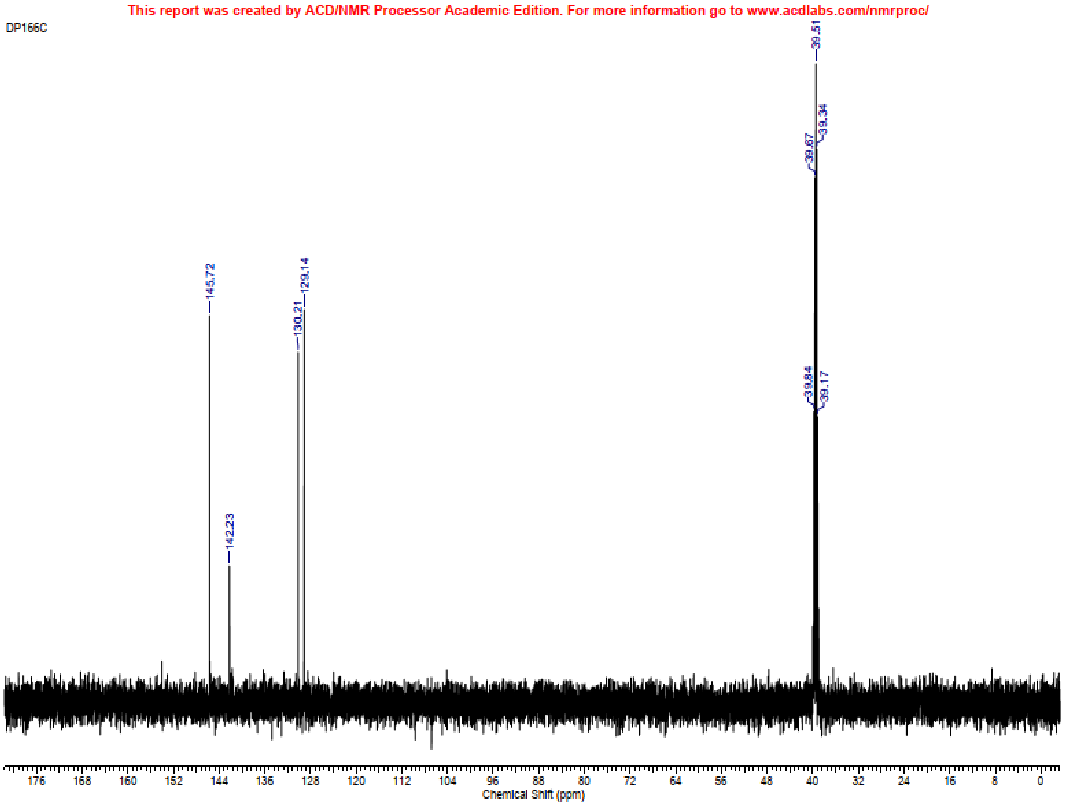

Figure S21. ^{1}H NMR spectrum of quinoxaline (above) and ^{13}C NMR spectrum (below).

6,7-dichloroquinoxaline (6c)

Yield = 99 %. **^{1}H NMR** (500 MHz, d-DMSO) δ 8.46 (2H, s, Ar*H*), 9.02 (2H, s, Ar*H*); **^{13}C NMR** (125 MHz, d-DMSO) δ 130.13, 133.01, 141.15, 147.13; **HRMS** calcd. for $C_8H_5Cl_2N_2$ $[M+H]^+$: 198.98234, found: 198.98252.

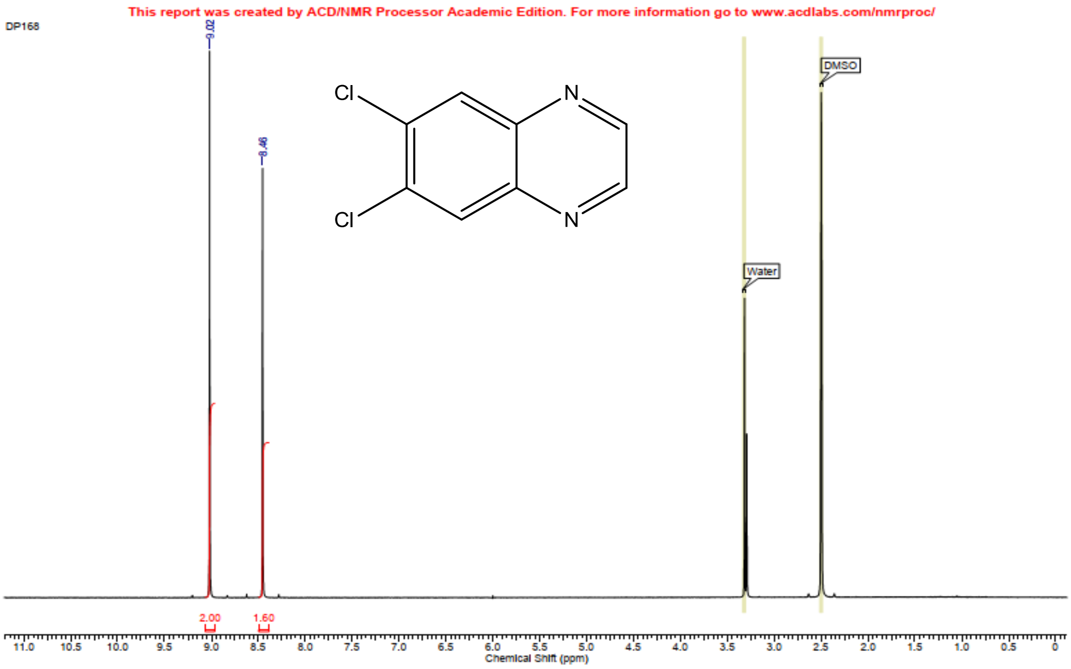

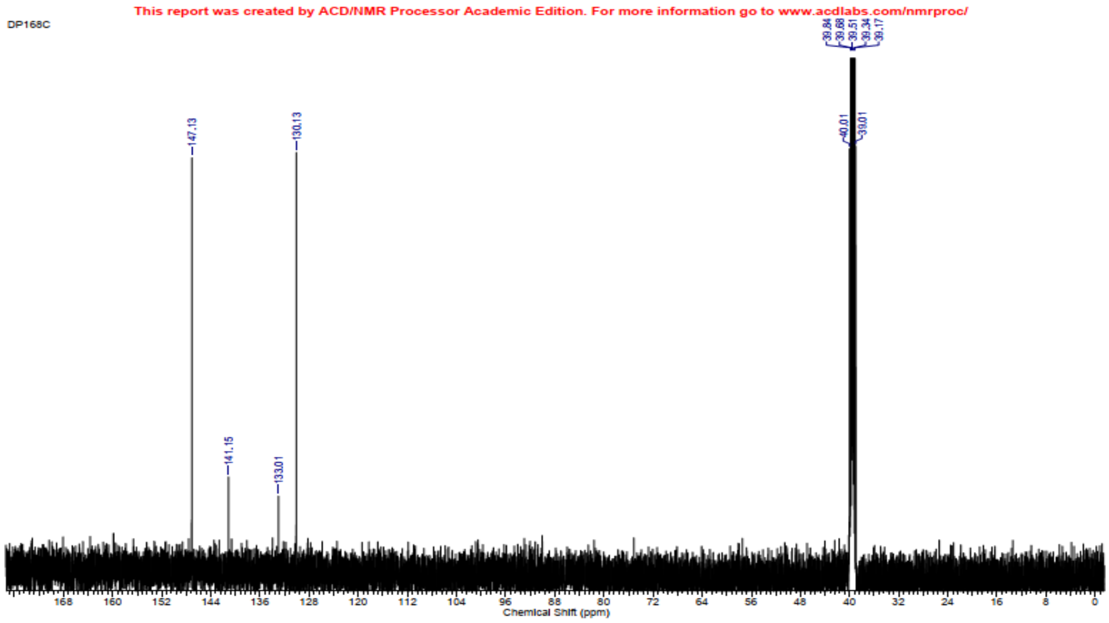

Figure S22. ^{1}H NMR spectrum of 6,7-dichloroquinoxaline (above) and ^{13}C NMR spectrum (below).

6,7-dibromoquinoxaline (6d)

Yield = 89 %. ^{1}**H NMR** (500 MHz, d-DMSO) δ 8.56 (2H, s, Ar*H*), 9.01 (2H, s, Ar*H*); ^{13}**C NMR** (125 MHz, d-DMSO) δ 125.77, 133.31, 141.56, 147.14; **HRMS** calcd. for $C_8H_5Br_2N_2$ $[M+H]^+$: 286.88140, found: 286.88165.

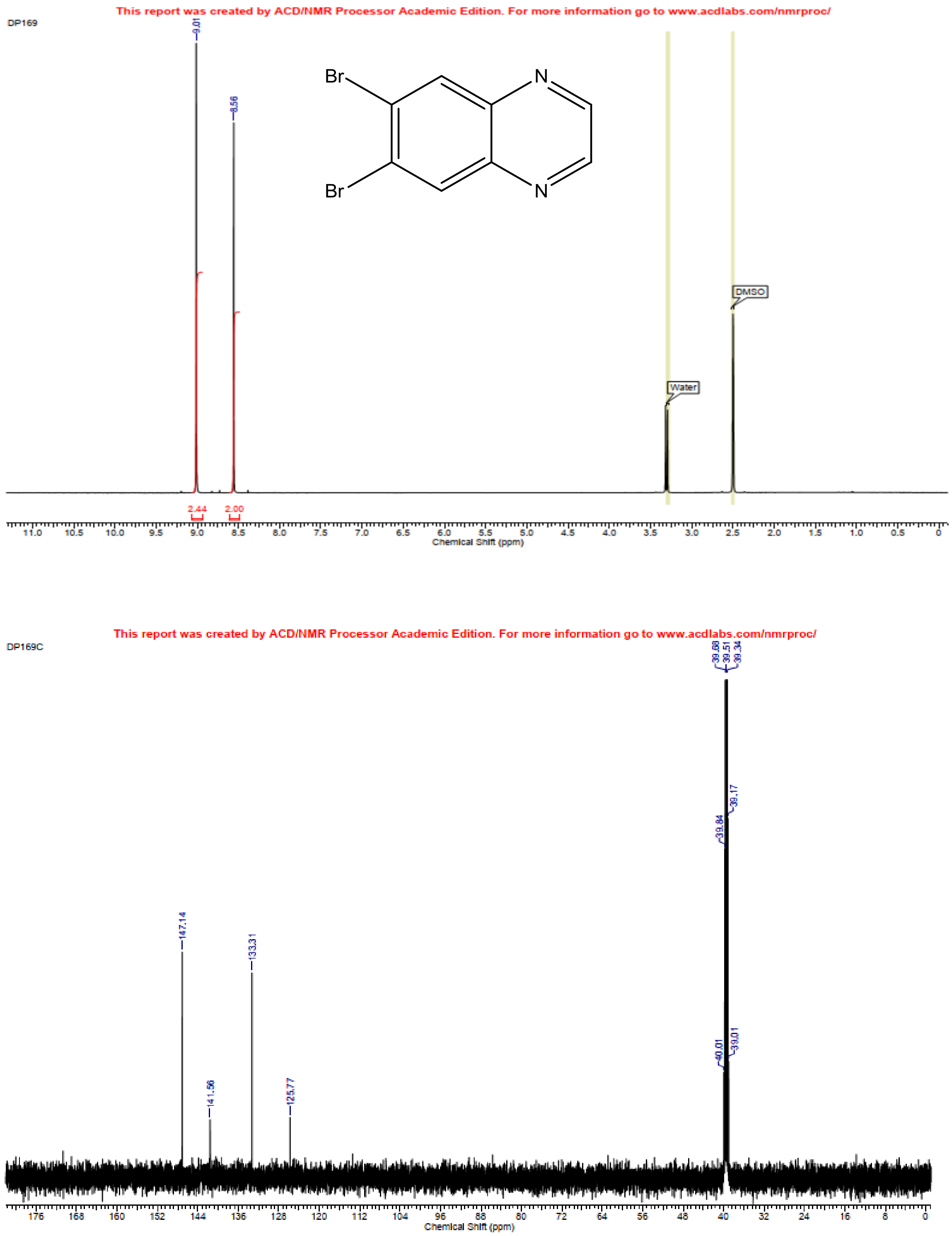

Figure S23. ^{1}H NMR spectrum of 6,7-dibromoquinoxaline (above) and ^{13}C NMR spectrum (below).

6,7-diiodoquinoxaline (6e)

Yield = 25 %. ^{1}**H NMR** (500 MHz, d-DMSO) δ 8.68 (2H, s, Ar*H*), 8.96 (2H, s, Ar*H*); ^{13}**C NMR** (125 MHz, d-DMSO) δ 111.06, 138.74, 141.92, 1467.83; **HRMS** calcd. for $C_8H_5I_2N_2$ $[M+H]^+$: 382.85366, found: 382.85426.

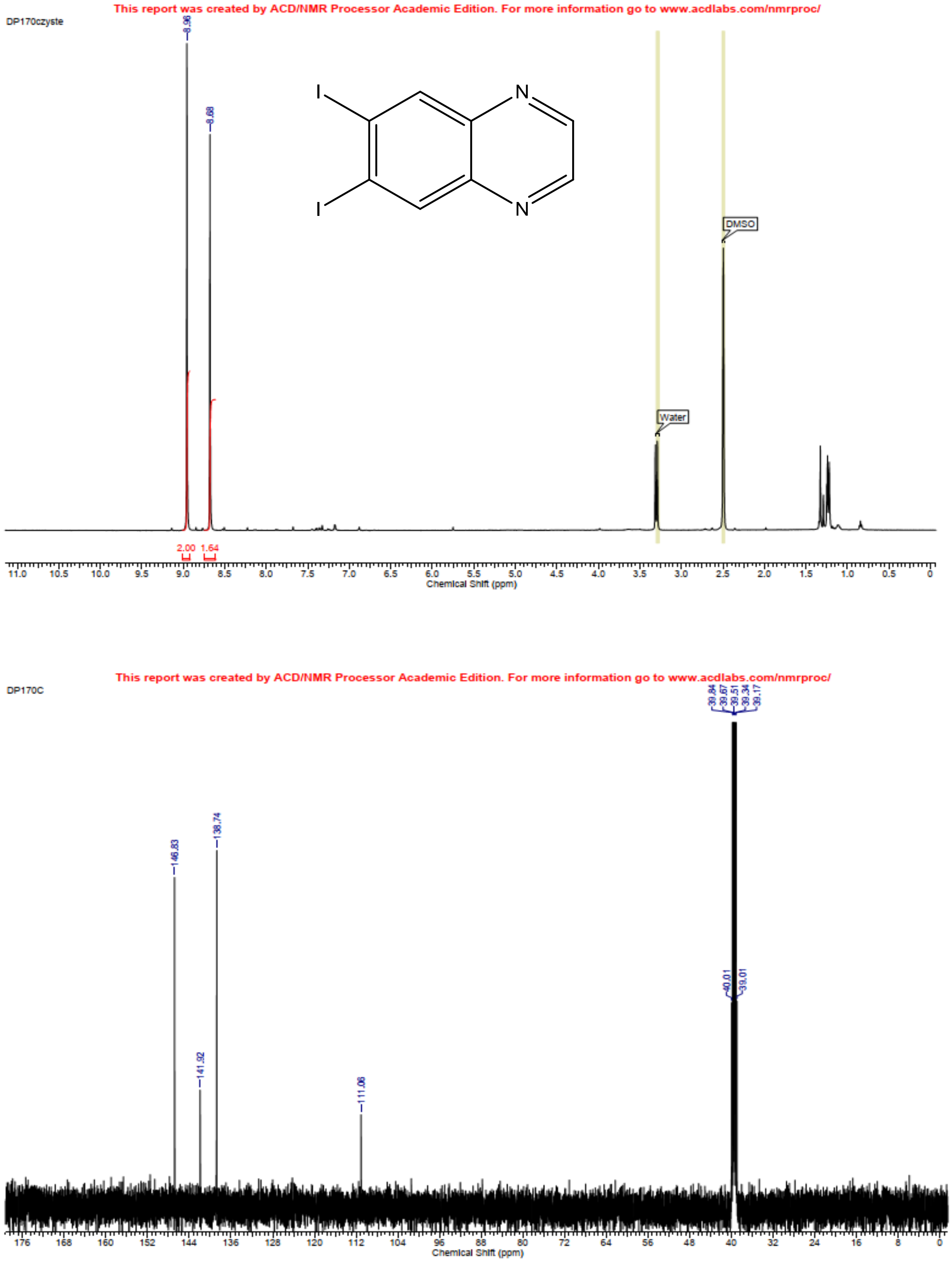

Figure S24. ^{1}H NMR spectrum of 6,7-diiodoquinoxaline (above) and ^{13}C NMR spectrum (below).

Synthesis of quinoxaline-2,3-diol derivatives (7a-e)

General procedure:

Benzene-1,2-diamine derivative (3 mmol) and oxalic acid (9 mmol, 810 mg) were heated at 120 °C in DMF (1 mL) overnight. The reaction was quenched by addition of water (20 mL). The participate was filtered and washed several times with water (compound **6a** was extracted with AcOEt (3x10 mL)). Crude products were purified by recrystallization from EtOH.

quinoxaline-1,3-diol (7a)

Yield = 57 %. ^{1}**H NMR** (500 MHz, d-DMSO) δ 7.02-7.10 (2H, m, Ar*H*), 7.10-7.16 (2H, m, Ar*H*), 11.90 (2H, s, 2xO*H*); ^{13}**C NMR** (125 MHz, d-DMSO) δ 115.12, 123.00, 125.60, 155.17; **HRMS** calcd. for $C_8H_7N_2O_2$ $[M+H]^+$: 163.05020, found: 163.05011.

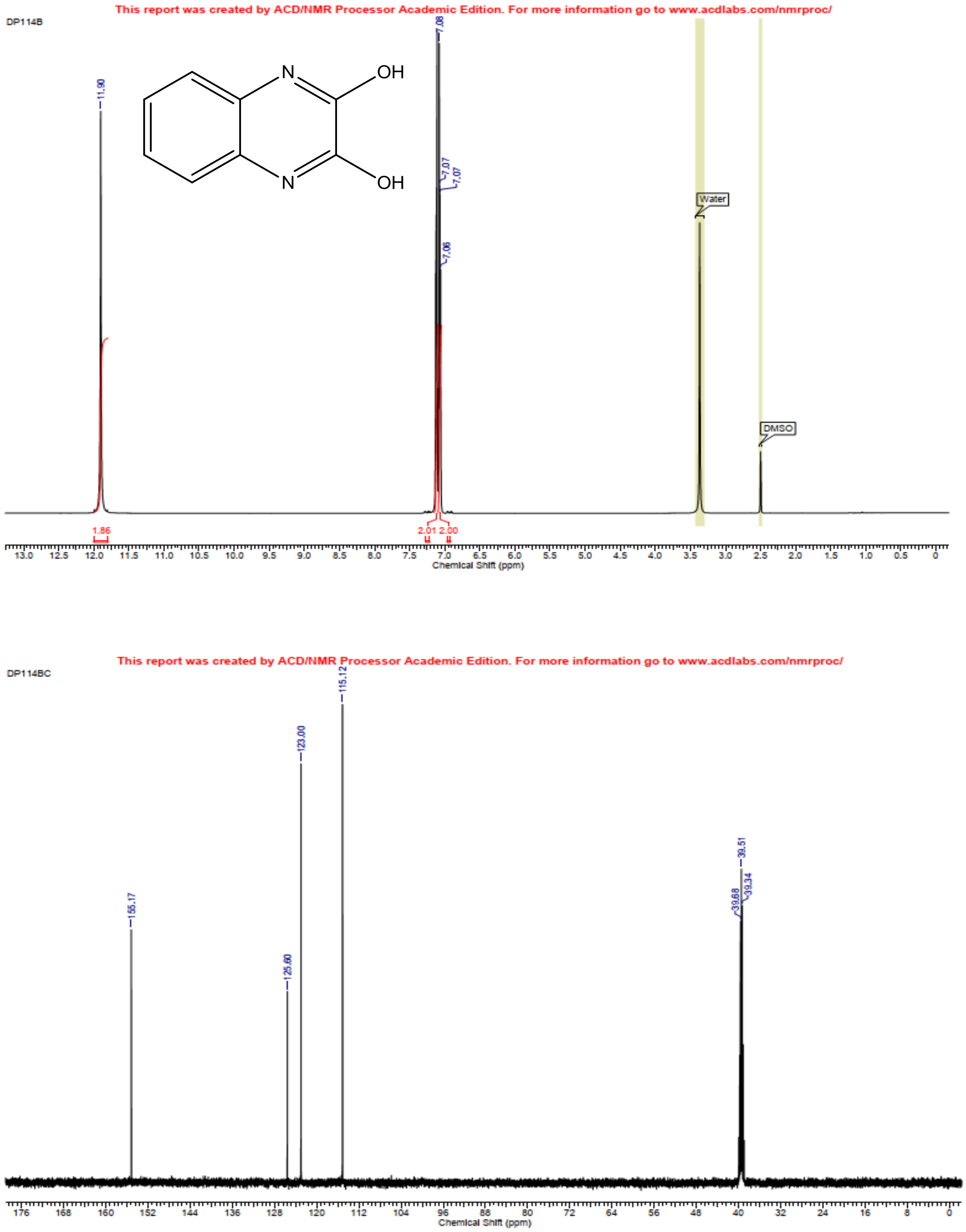

Figure S25. ^{1}H NMR spectrum of quinoxaline-2,3-diol (above) and ^{13}C NMR spectrum (below).

6,7-difluoroquinoxaline-1,3-diol (7b)

Yield = 68 %. **^{1}H NMR** (500 MHz, d-DMSO) δ 7.00-7.11 (2H, m, Ar*H*), 11.92 (2H, s2xO*H*); **^{13}C NMR** (125 MHz, d-DMSO) δ 103.63, 103.74, 103.81, 122.26, 122.32, 143.91, 144.04, 145.83, 145.96, 154.70; **HRMS** calcd. for $C_8H_5F_2N_2O_2$ $[M+H]^+$: 199.03136, found: 199.03138.

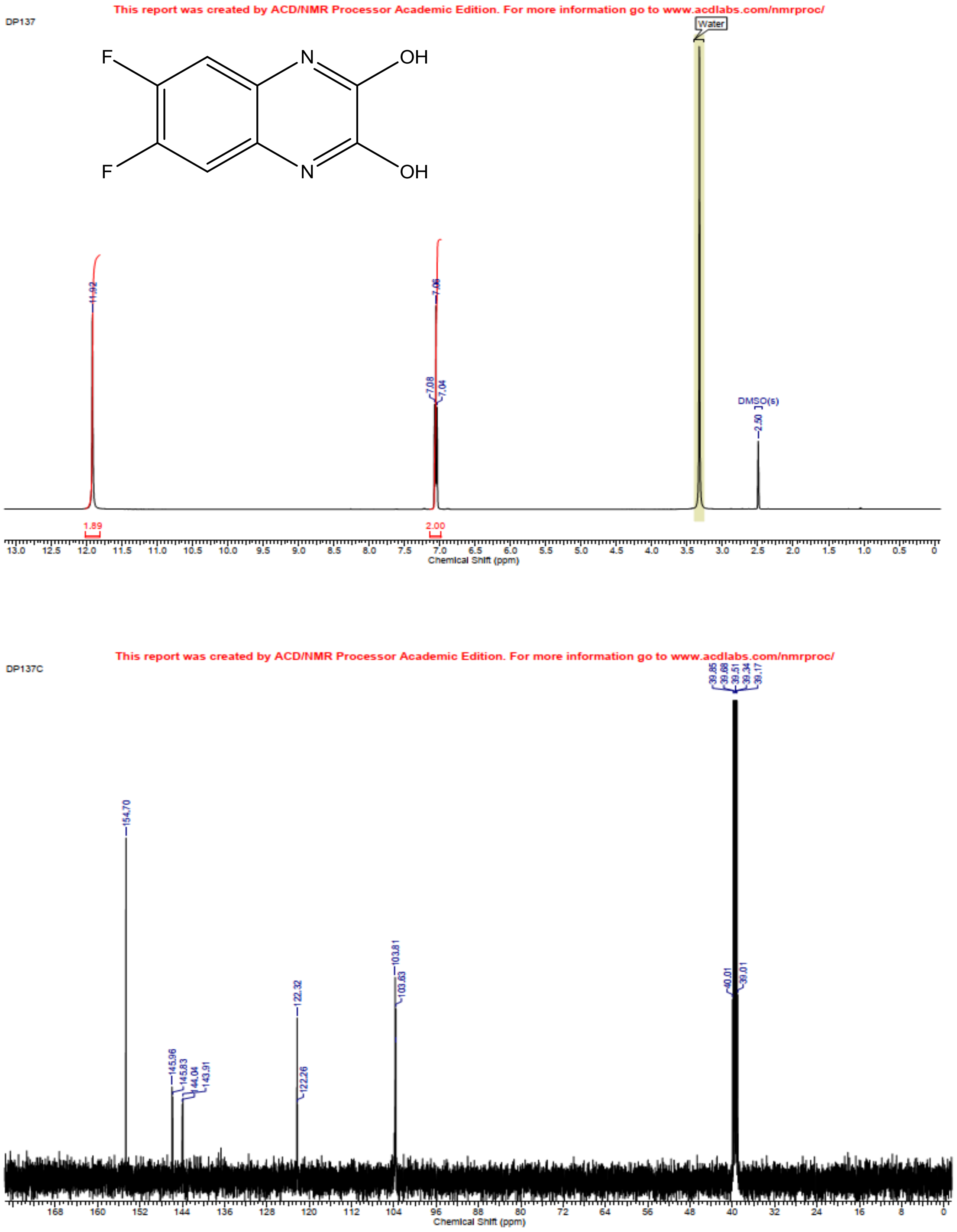

Figure S26. ^{1}H NMR spectrum of 6,7-difluoroquinoxaline-2,3-diol (above) and ^{13}C NMR spectrum (below).

6,7-dichloroquinoxaline-1,3-diol (7c)

Yield = 86 %. [1]**H NMR** (500 MHz, d-DMSO) δ 7.21 (2H, s, Ar*H*), 12.00 (2H, s, 2xO*H*); [13]**C NMR** (100 MHz, d-DMSO) δ 115.99, 124.29, 126.03, 154.71; **HRMS** calcd. for $C_8H_5Cl_2N_2O_2$ $[M+H]^+$: 230.97226, found: 230.97218.

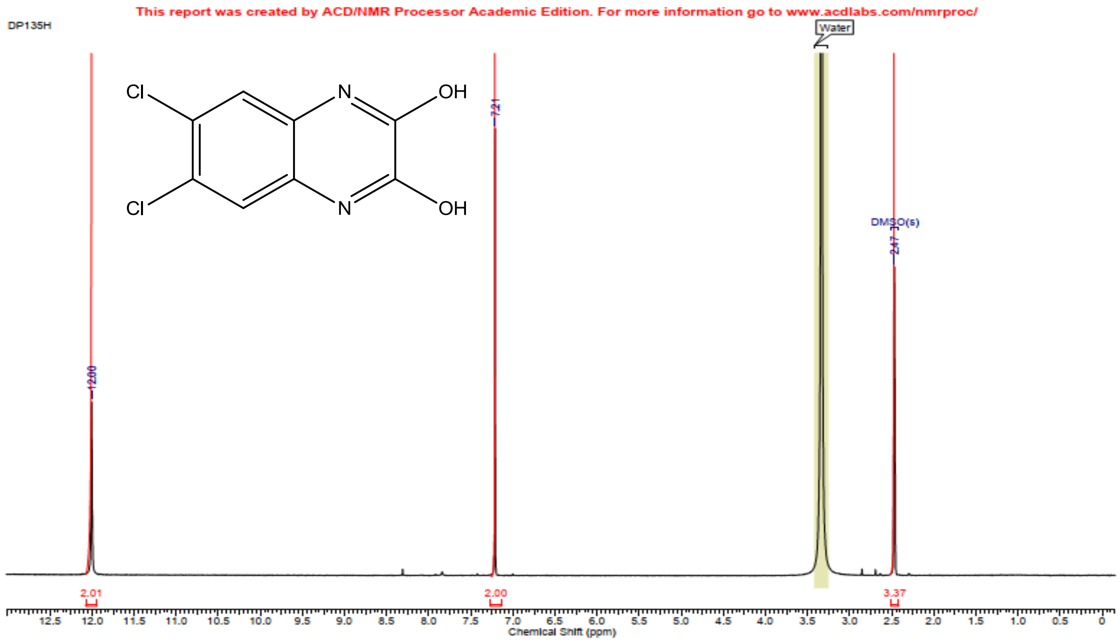

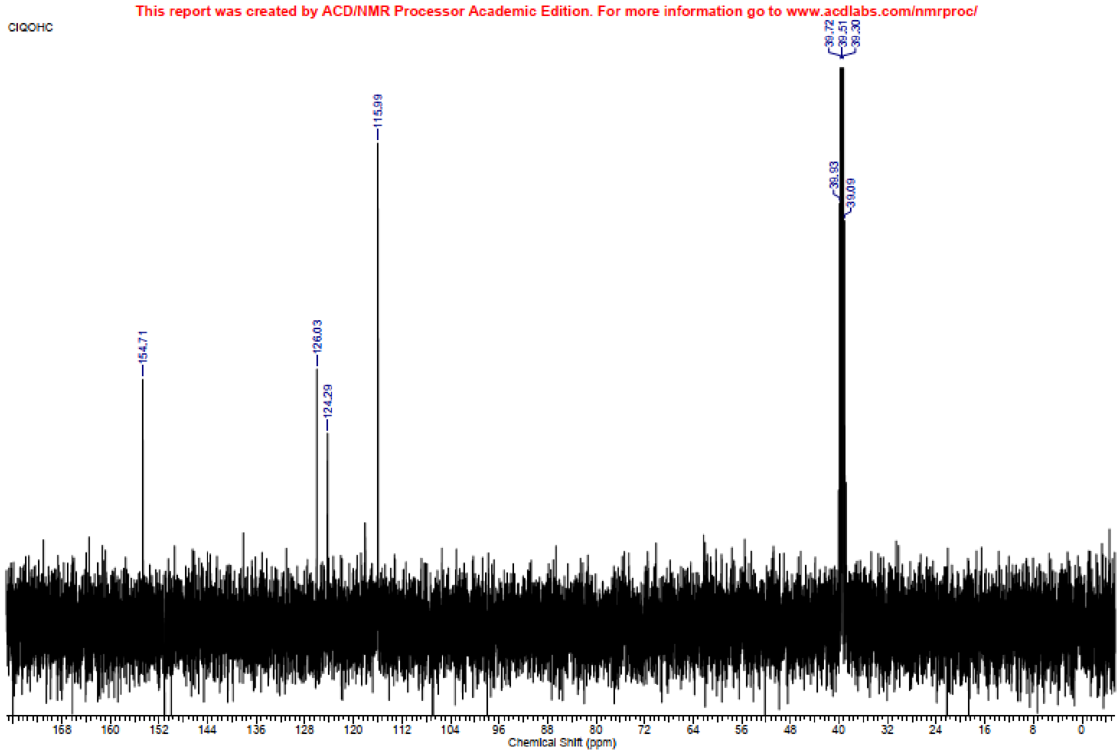

Figure S27. ^{1}H NMR spectrum of 6,7-dichloroquinoxaline-2,3-diol (above) and ^{13}C NMR spectrum (below).

6,7-dibromooquinoxaline-1,3-diol (7d)

Yield = 91 %. ^{1}**H NMR** (500 MHz, d-DMSO) δ 7.38 (2H, s, Ar*H*), 11.99 (2H, s, 2xO*H*); ^{13}**C NMR** (100 MHz, d-DMSO) δ 116.15, 118.96, 126.64, 154.73; **HRMS** calcd. for $C_8H_5Br_2N_2O_2$ $[M+H]^+$: 318.87123, found: 318.87105.

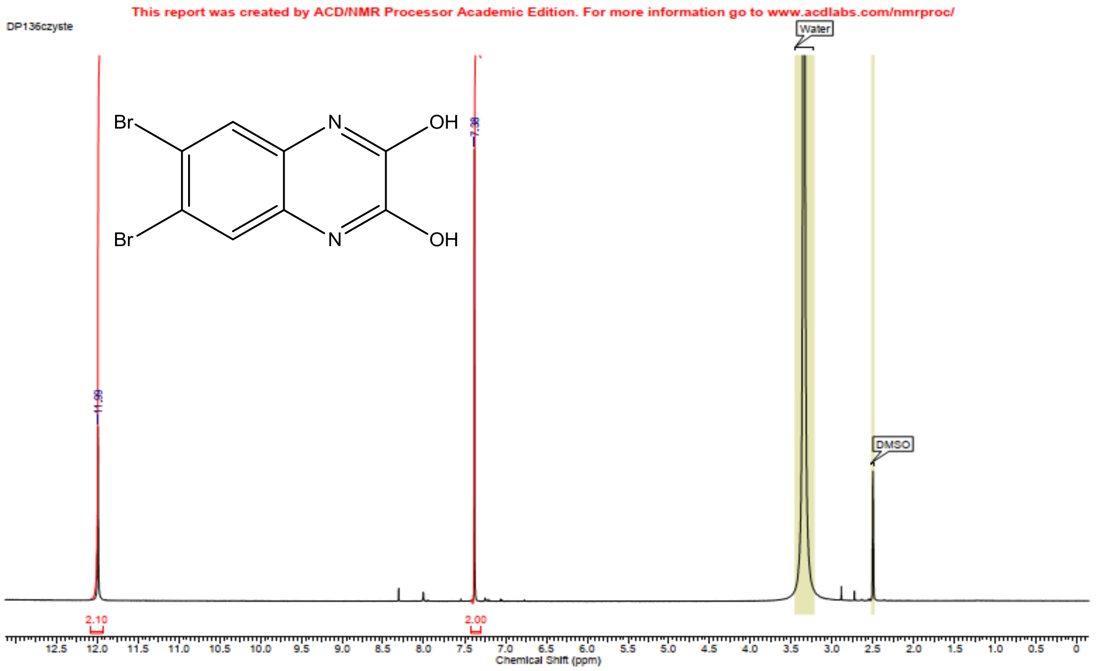

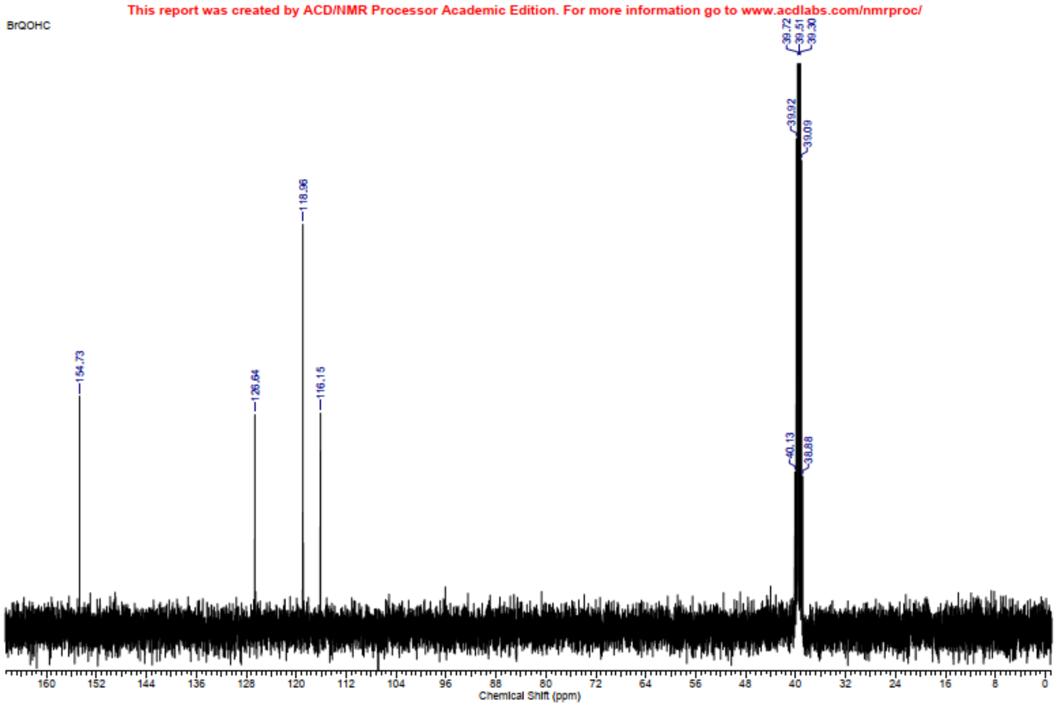

Figure S28. ^{1}H NMR spectrum of 6,7-dibromoquinoxaline-2,3-diol (above) and ^{13}C NMR spectrum (below).

6,7-diiodooquinoxaline-1,3-diol (7e)

Yield = 63 %. [1]**H NMR** (400 MHz, d-DMSO) δ 7.51 (2H, s, Ar*H*), 11.77 (2H, s br, 2xO*H*); [13]**C NMR** (100 MHz, d-DMSO) δ 99.11, 124.80, 128.06, 155.49; **HRMS** calcd. for $C_8H_5I_2N_2O_2$ $[M+H]^+$: 414.84349, found: 414.84366.

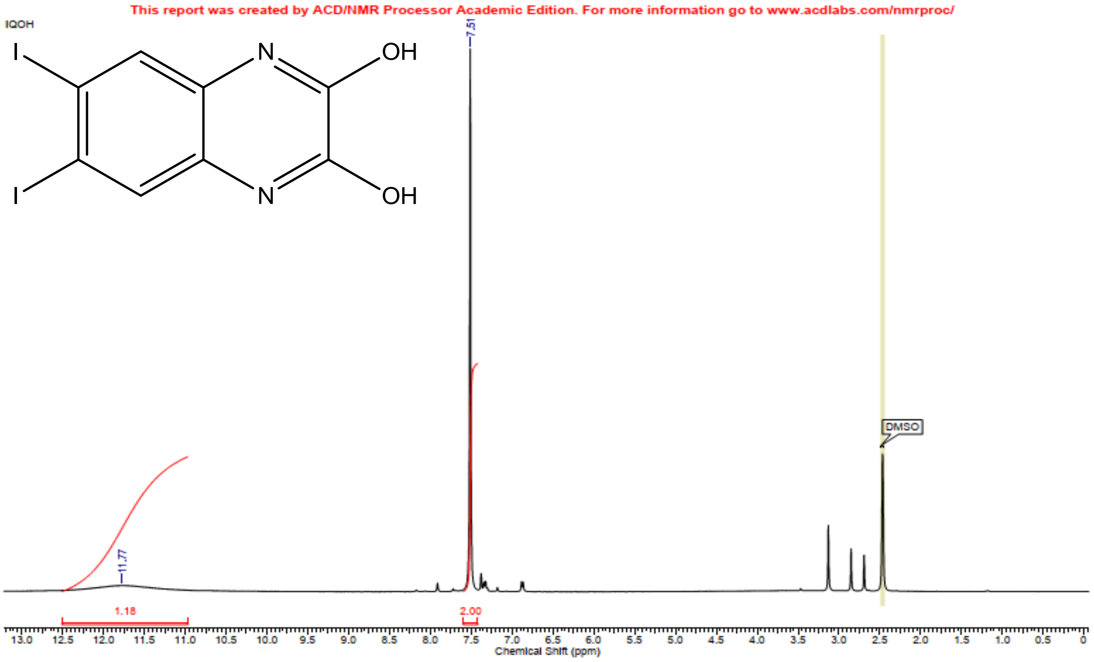

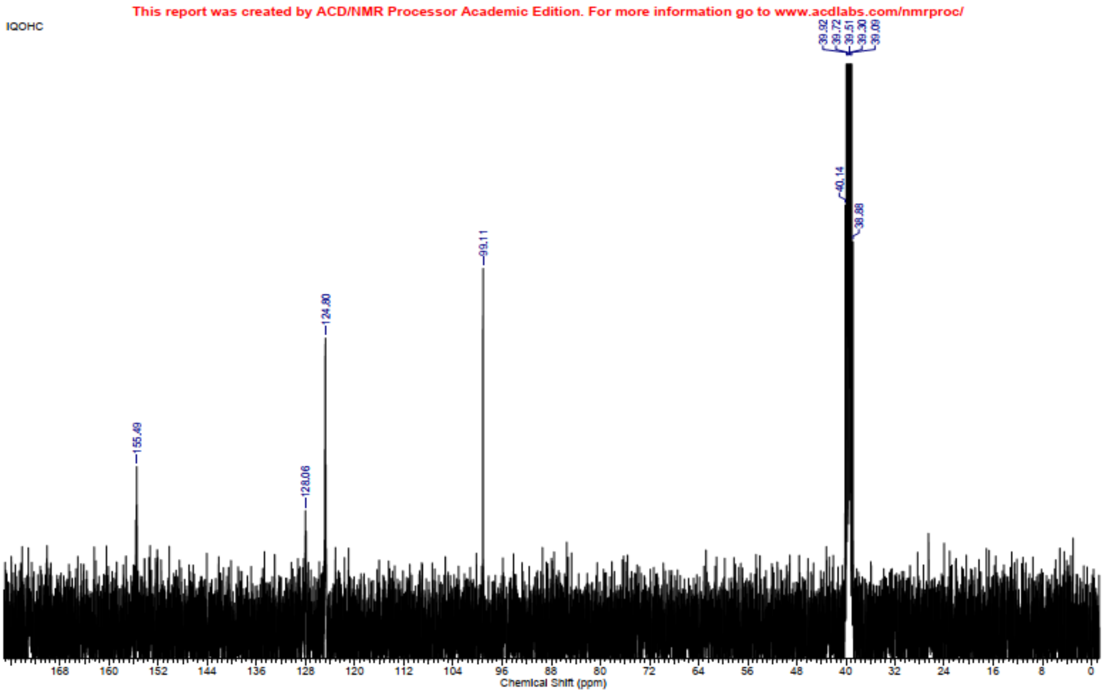

Figure S29. ^{1}H NMR spectrum of 6,7-diiodoquinoxaline-2,3-diol (above) and ^{13}C NMR spectrum (below).

2. Cell viability

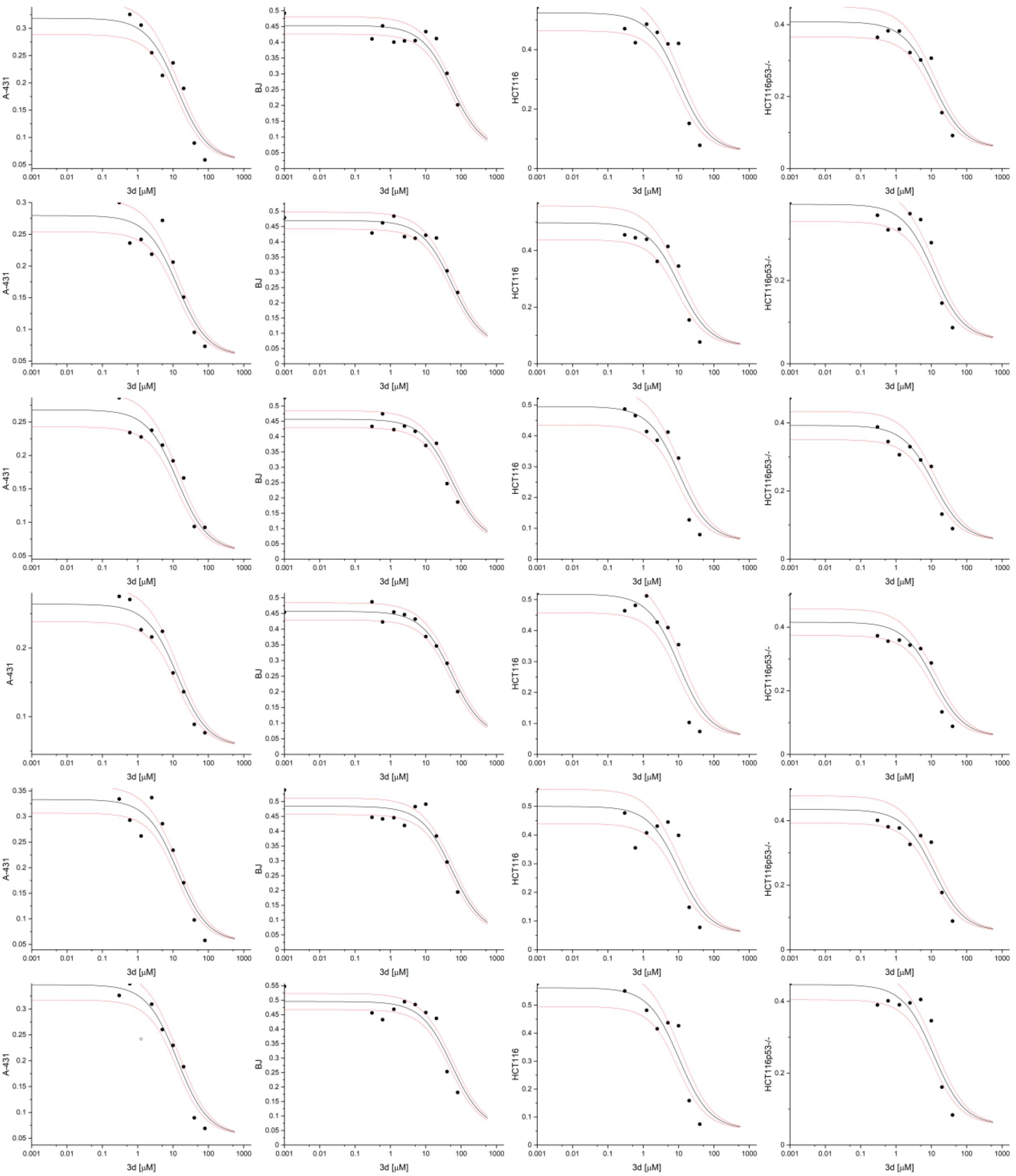

Figure S30 Viability of four selected cell lines in the presence of compound **3d**. Each column shows six independent experiments (A-431, BJ, HTC116 and HTC116p53$^{-/-}$ cell lines, respectively), solid circles denotes experimental data, solid lines follow the model fitted globally to six experiments, while dashed lines board 95% confidence bands for the model.

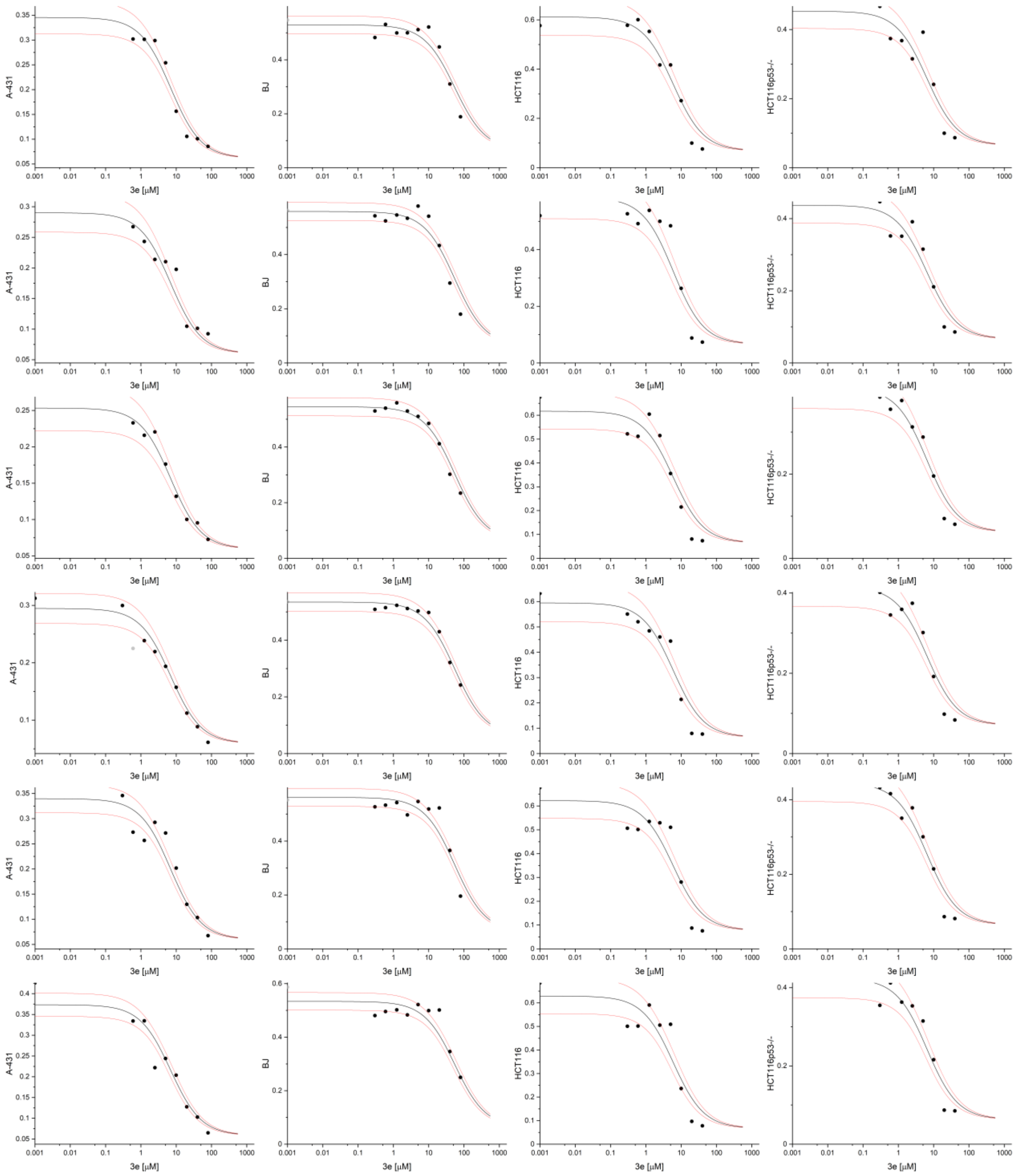

Figure S31 Viability of four selected cell lines in the presence of compound **3e**. Each column shows six independent experiments (A-431, BJ, HTC116 and HTC116p53$^{-/-}$ cell lines, respectively), solid circles denotes experimental data, solid lines follow the model fitted globally to six experiments, while dashed lines board 95% confidence bands for the model.

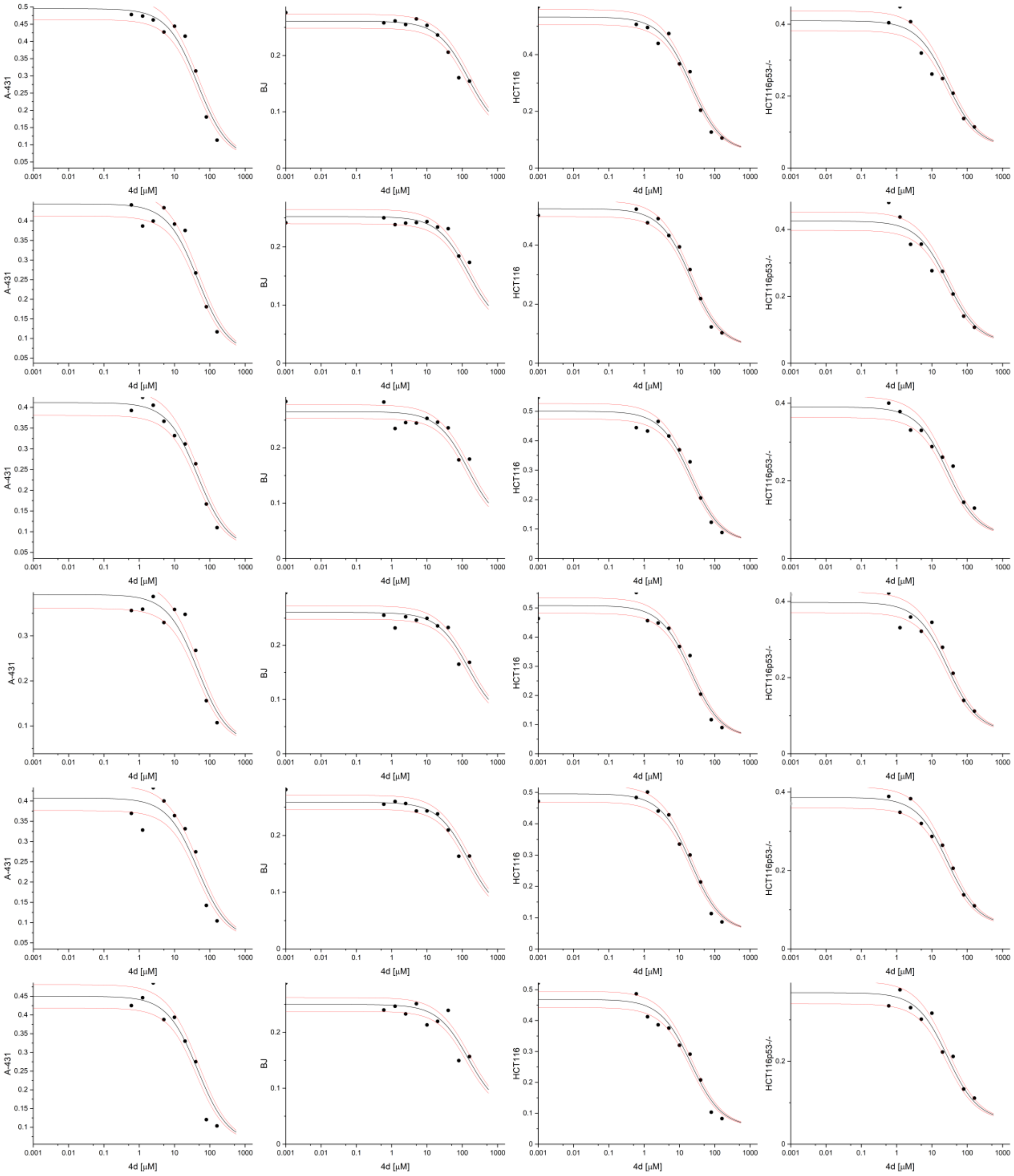

Figure S32 Viability of four selected cell lines in the presence of compound **4d**. Each column shows six independent experiments (A-431, BJ, HTC116 and HTC116p53$^{-/-}$ cell lines, respectively), solid circles denotes experimental data, solid lines follow the model fitted globally to six experiments, while dashed lines board 95% confidence bands for the model.

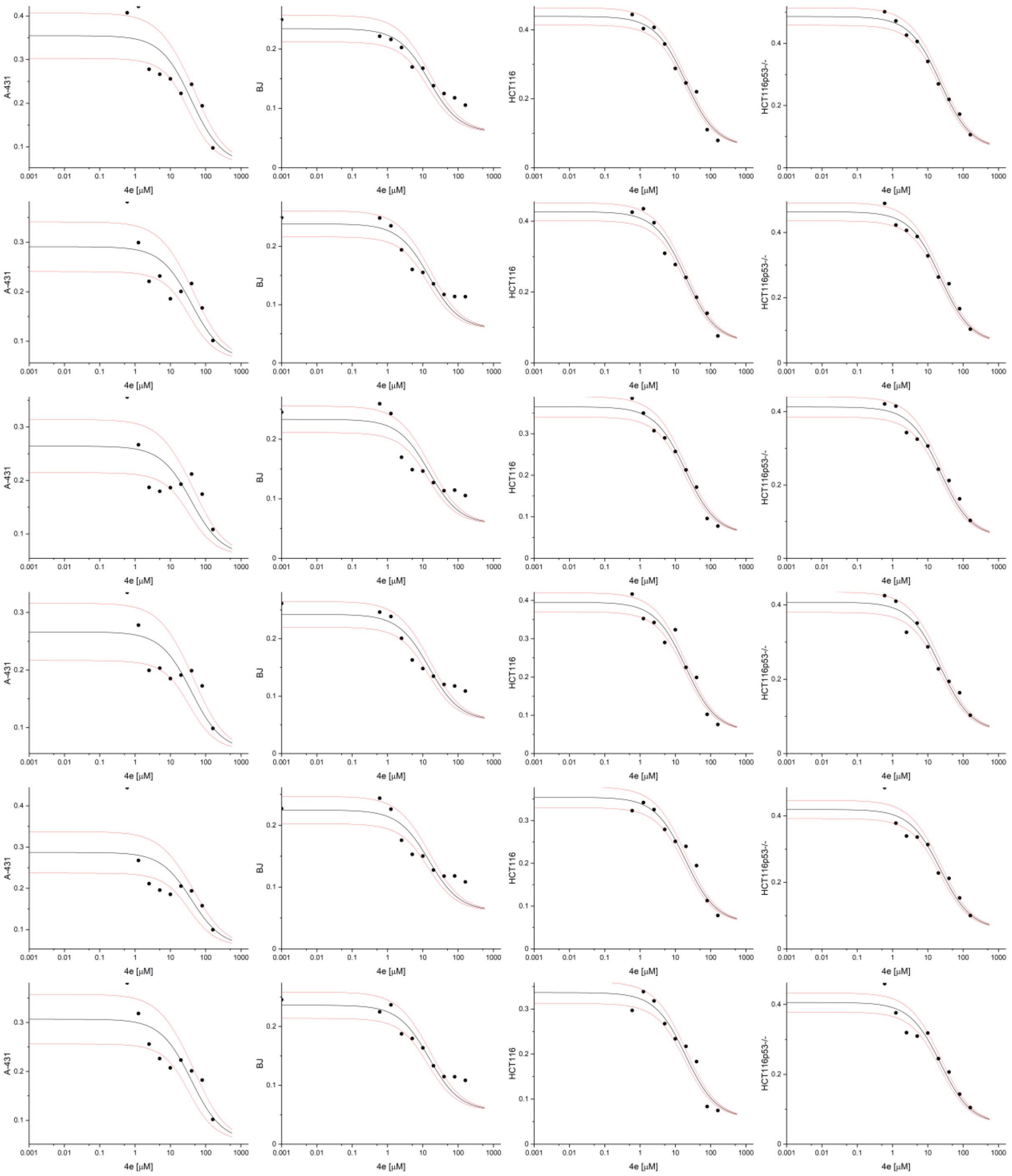

Figure S33 Viability of four selected cell lines in the presence of compound **4e**. Each column shows six independent experiments (A-431, BJ, HTC116 and HTC116p53$^{-/-}$ cell lines, respectively), solid circles denotes experimental data, solid lines follow the model fitted globally to six experiments, while dashed lines board 95% confidence bands for the model.

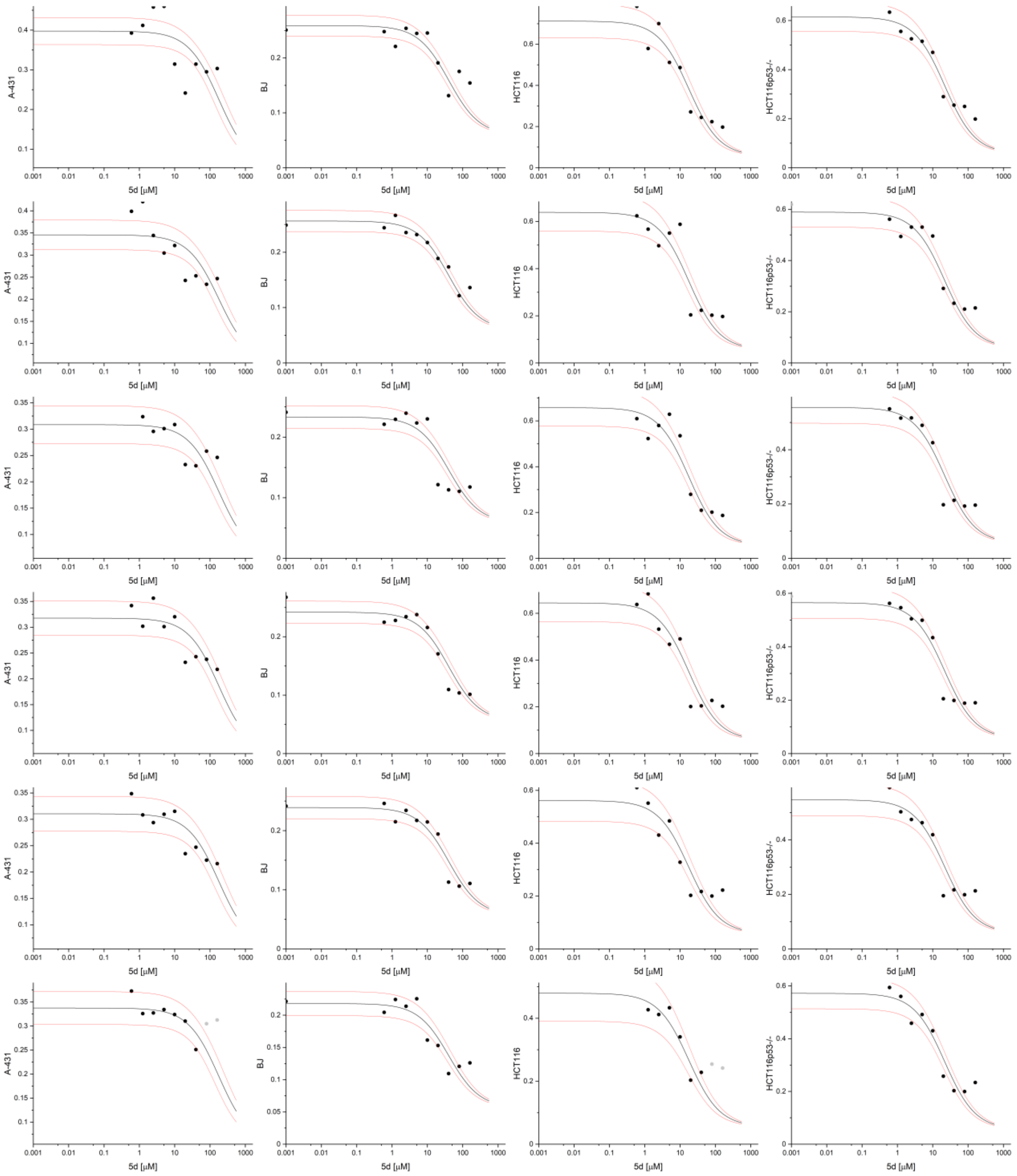

Figure S34 Viability of four selected cell lines in the presence of compound 5**d**. Each column shows six independent experiments (A-431, BJ, HTC116 and HTC116p53$^{-/-}$ cell lines, respectively), solid circles denotes experimental data, solid lines follow the model fitted globally to six experiments, while dashed lines board 95% confidence bands for the model.

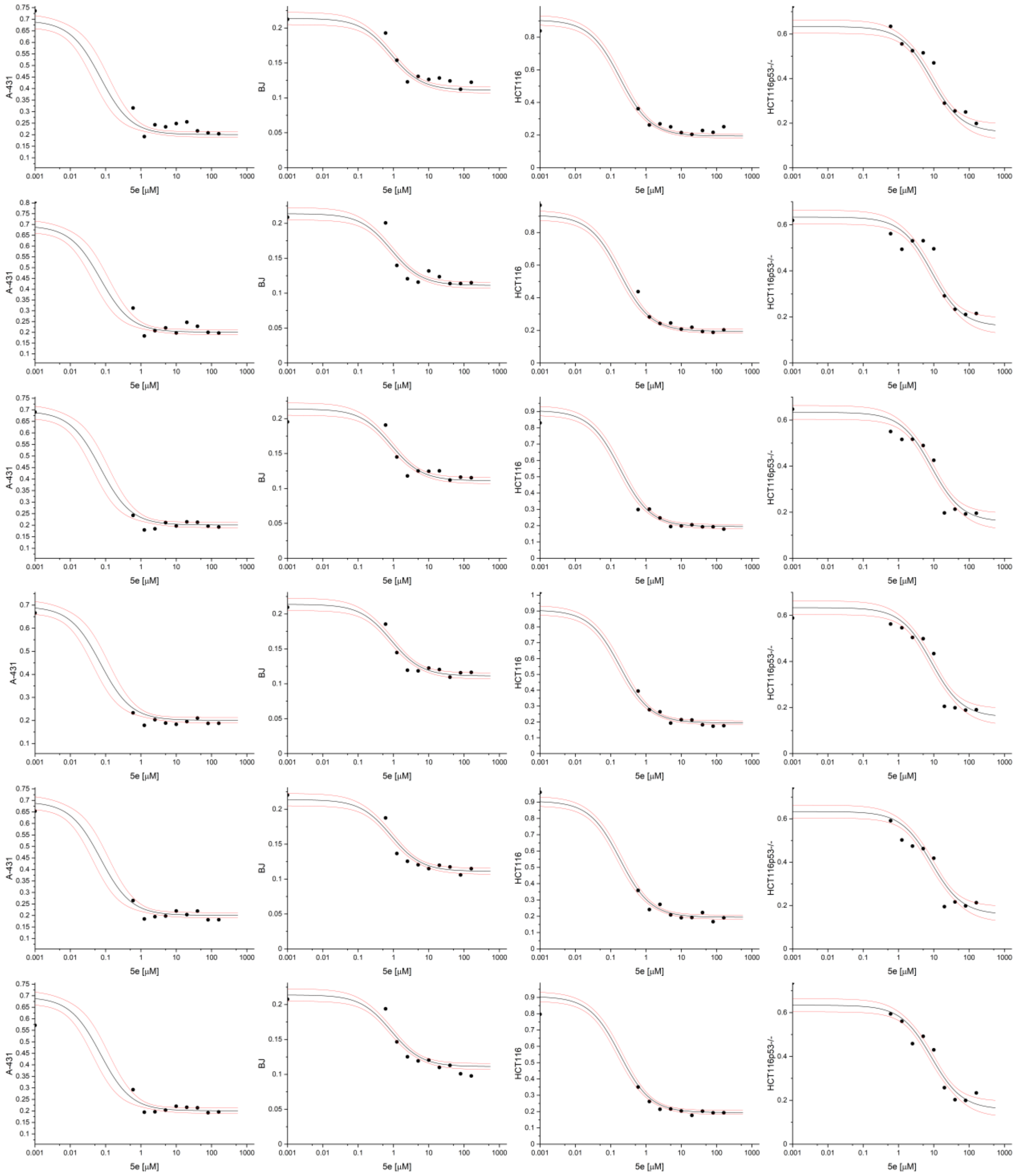

Figure S35 Viability of four selected cell lines in the presence of compound **5e**. Each column shows six independent experiments (A-431, BJ, HTC116 and HTC116p53$^{-/-}$ cell lines, respectively), solid circles denotes experimental data, solid lines follow the model fitted globally to six experiments, while dashed lines board 95% confidence bands for the model.

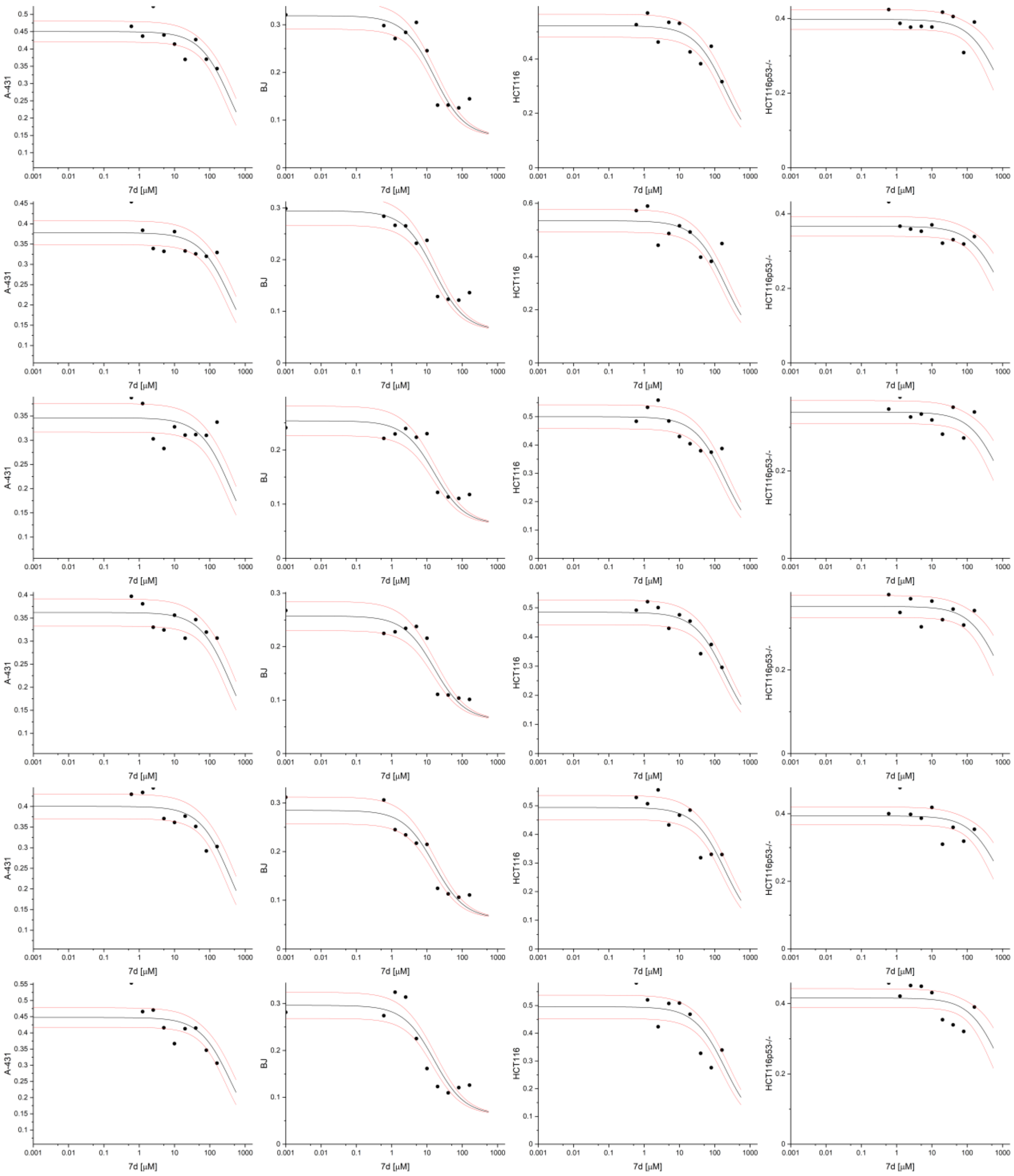

Figure S36 Viability of four selected cell lines in the presence of compound **7d**. Each column shows six independent experiments (A-431, BJ, HTC116 and HTC116p53$^{-/-}$ cell lines, respectively), solid circles denotes experimental data, solid lines follow the model fitted globally to six experiments, while dashed lines board 95% confidence bands for the model.

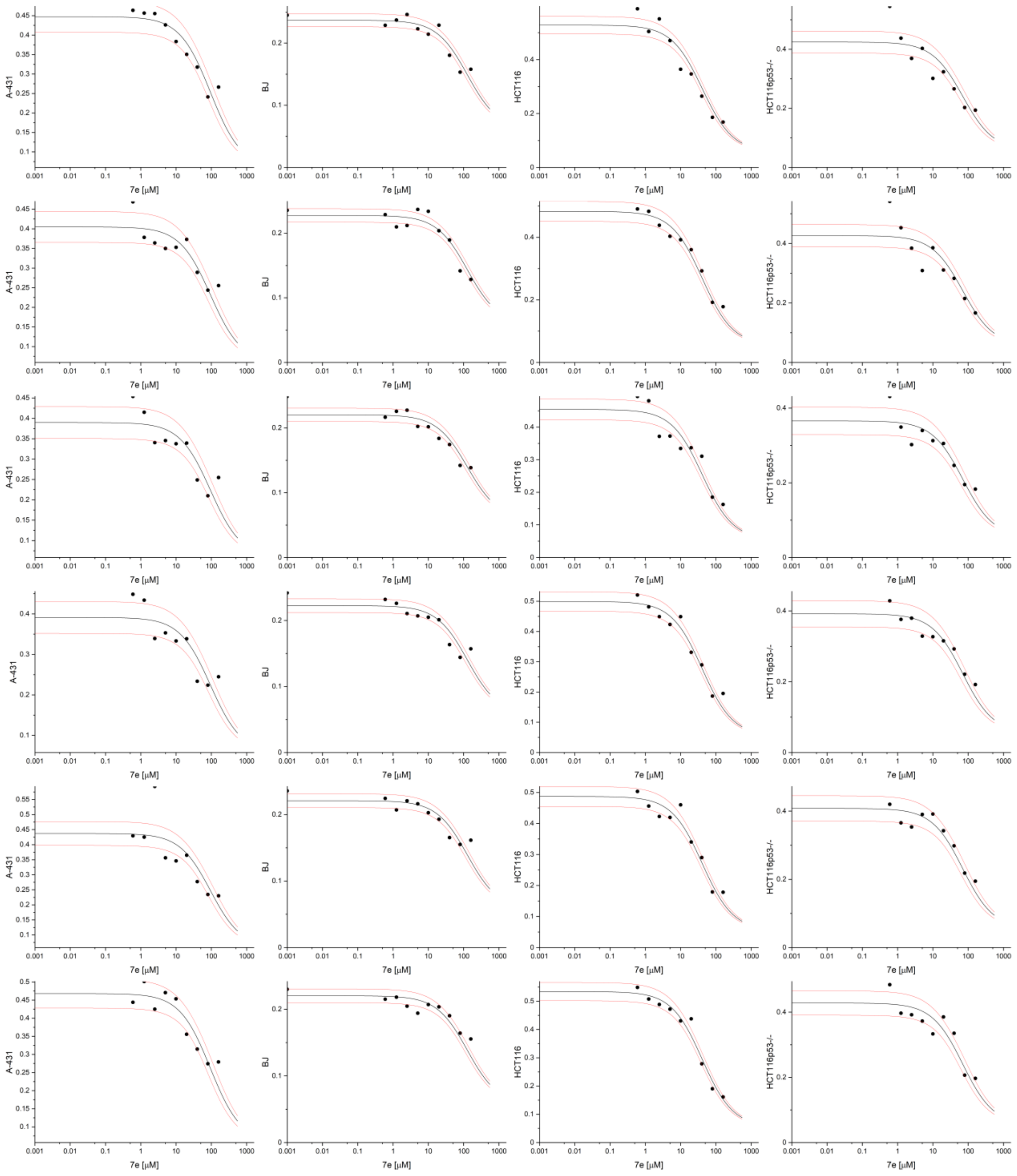

Figure S37 Viability of four selected cell lines in the presence of compound **7e**. Each column shows six independent experiments (A-431, BJ, HTC116 and HTC116p53$^{-/-}$ cell lines, respectively), solid circles denotes experimental data, solid lines follow the model fitted globally to six experiments, while dashed lines board 95% confidence bands for the model.

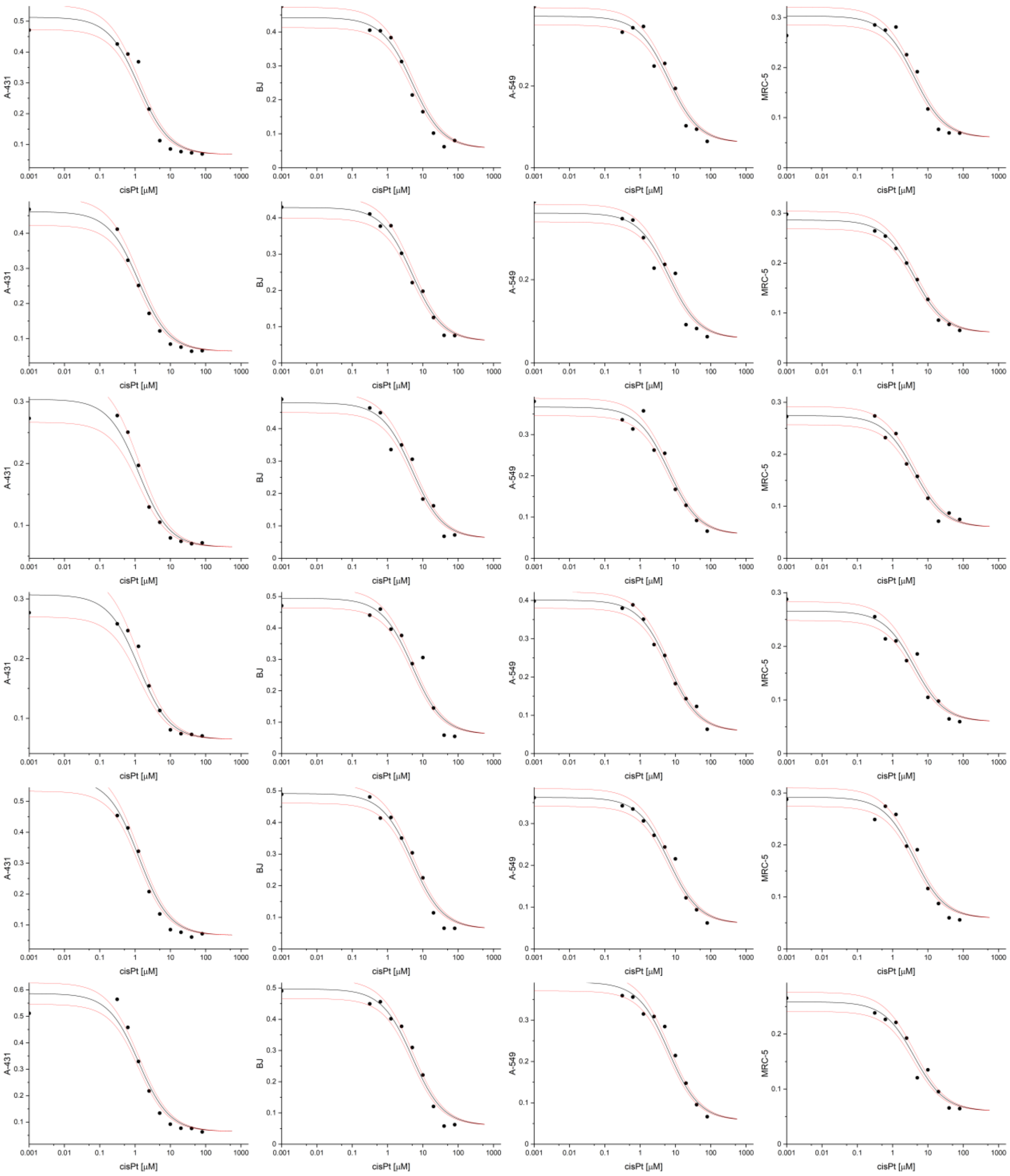

Figure S37 Viability of four selected cell lines in the presence of cisplatin. Each column shows six independent experiments (A-431, BJ, HTC116 and HTC116p53$^{-/-}$ cell lines, respectively), solid circles denotes experimental data, solid lines follow the model fitted globally to six experiments, while dashed lines board 95% confidence bands for the model.

3. Enzymatic in vitro activity.

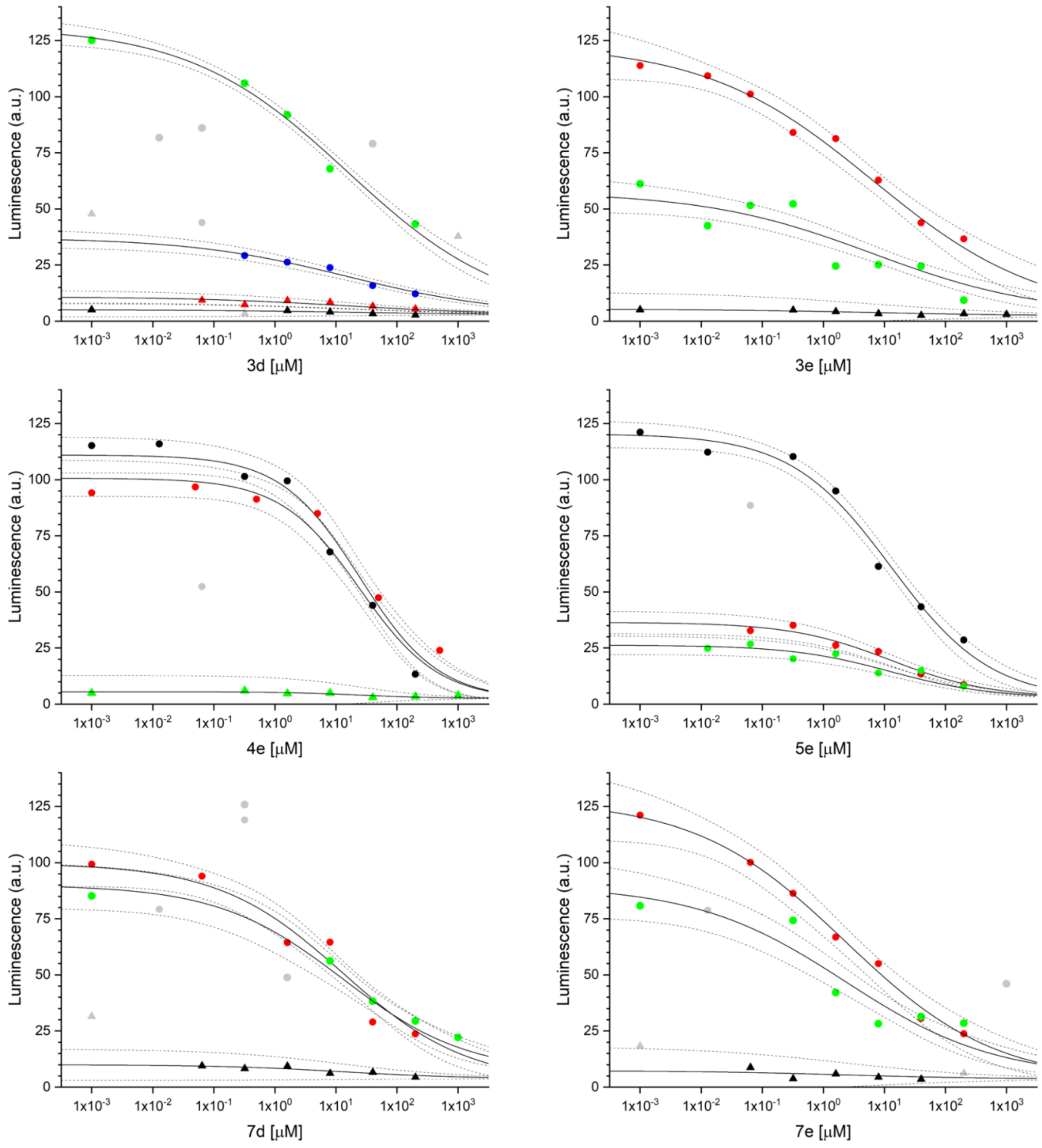

Figure S38 The inhibition of hCK2α by selected compounds. Gray circles indicate data excluded from the global analysis of at least three independent experiments.

4. Molecular modeling data

Table S1. Short statistics concerning ligand location at the ATP-binding site of protein kinase CK2 in low-energy complexes (ΔG_{bind} less than 0.5 kcal/mol above the lowest-energy complex). Location of the ligand was screened by the distance to Nζ of Lys 68 (i.e. salt bridge promoting location) and by the distance between a halogen atom and the backbone carbonyl oxygen of Glu114 and Val116, both of which are the most abundant X-bond acceptors identified in CK2 complexes deposed in PDB. The minimal, maximal, median and quartile values are listed.

ligand	Distance [Å]									
	Ligand to Nζ of Lys68					X of ligand to backbone O of the hinge residue				
	min	Q1	median	Q3	max	min	Q1	median	Q3	max
**1b**	2.89	3.03	3.40	3.94	4.76	3.11	3.54	3.72	4.11	6.53
**1c**	3.11	3.52	3.56	4.06	5.80	3.87	4.47	5.73	7.30	8.38
**1d**	3.71	4.20	4.58	5.01	6.54	4.64	5.82	7.60	8.12	8.37
**1e**	4.45	4.68	4.88	5.07	6.55	4.85	5.26	5.90	6.80	8.10
**2b**	3.03	3.17	4.04	4.49	5.29	3.76	4.13	5.37	6.56	6.81
**2c**	2.79	2.99	3.70	3.83	4.44	3.68	4.39	5.03	5.82	6.95
**2d**	2.75	2.80	2.93	3.41	3.58	3.66	3.74	4.61	5.84	7.00
**2e**	2.70	2.91	3.69	3.81	4.72	3.73	4.00	4.82	5.64	5.89
**3b**	3.67	3.67	3.98	4.36	5.10	3.13	3.79	6.60	8.37	9.58
**3c**	2.94	3.20	3.65	3.79	3.79	3.04	4.60	6.98	10.54	11.94
**3d**	2.76	3.09	3.09	4.00	4.00	2.94	3.71	6.76	11.74	12.27
**3e**	3.60	3.66	3.84	4.35	4.77	3.82	5.49	11.93	12.29	12.56
**4b**	4.16	4.19	4.64	4.88	5.46	3.55	3.74	5.20	6.62	6.84
**4c**	2.85	2.94	3.37	4.08	4.73	3.66	3.96	6.40	6.80	7.26
**4d**	3.02	3.02	3.06	5.22	5.61	3.77	3.84	4.87	6.79	6.91
**4e**	2.86	2.94	3.70	5.20	5.56	3.87	4.11	4.94	5.98	6.82
**5b**	2.99	3.60	3.62	4.47	5.28	3.76	4.26	6.43	6.76	8.71
**5c**	3.11	3.12	3.15	3.15	3.25	3.42	3.50	4.98	6.44	6.49
**5d**	2.75	2.99	2.99	3.09	3.60	3.64	3.65	4.77	5.66	6.79
**5e**	2.72	2.94	2.94	3.11	3.61	3.74	3.93	4.87	5.76	7.15
**6b**	2.92	3.02	3.59	4.02	4.02	3.94	3.99	6.44	6.65	8.42
**6c**	2.81	3.57	3.86	4.23	4.52	3.67	4.47	6.14	6.97	8.69
**6d**	2.73	2.74	2.76	3.35	3.58	3.64	3.82	4.74	5.67	5.90
**6e**	3.06	3.26	3.85	3.86	4.80	3.82	3.98	4.77	5.65	6.02
**7b**	4.00	4.00	4.00	4.36	4.71	3.88	3.88	5.24	6.59	6.59
**7c**	2.29	2.42	2.81	3.04	3.54	3.32	3.61	4.15	4.87	5.67
**7d**	2.79	2.89	3.45	3.73	3.73	3.57	4.94	7.20	9.28	10.09
**7e**	2.32	2.45	2.82	2.87	3.19	3.80	3.81	4.33	5.01	5.48

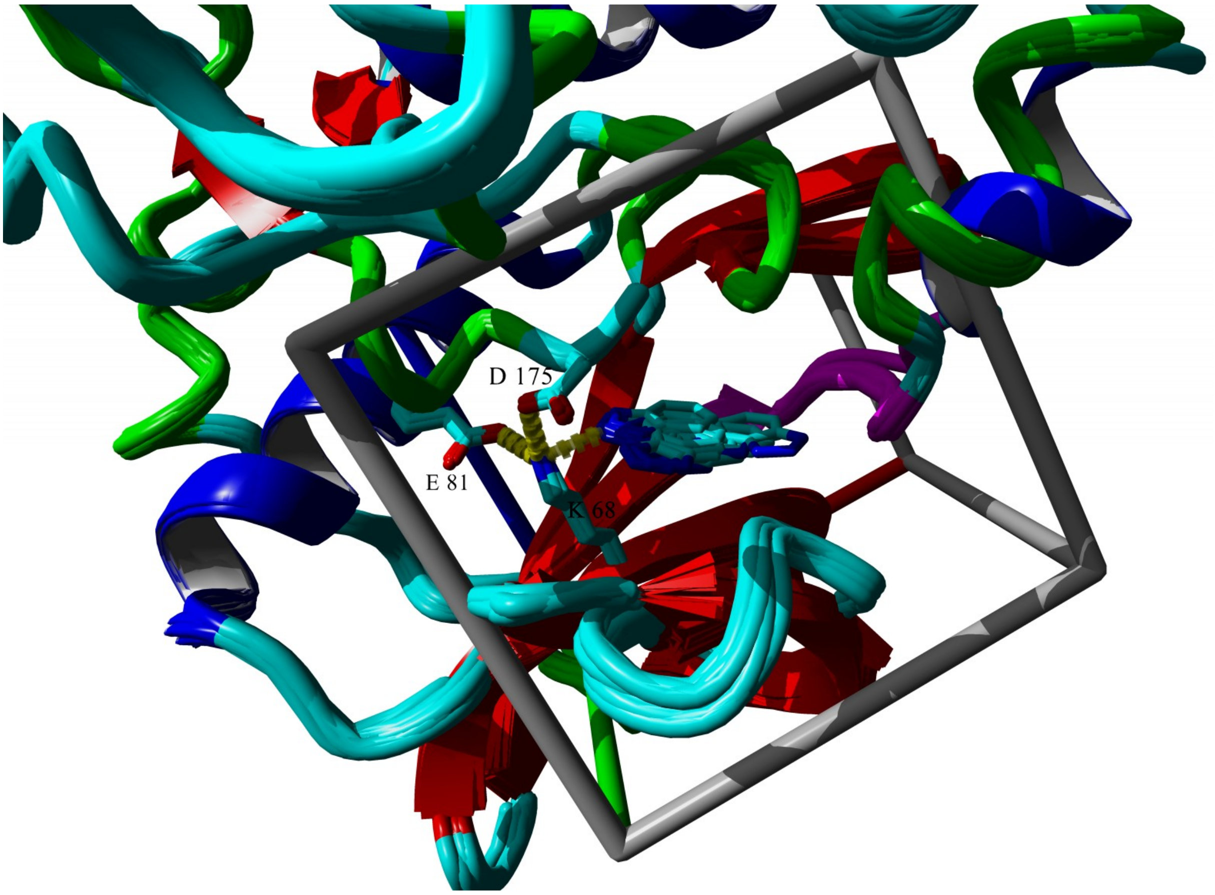

Figure S39. Location of ligand in structures used as templates in molecular docking procedure (6TLW, 6TLV, 6TLU, 6TLS, 6TLR, 6TLP, 6TLO and 6TLL). Hydrogen bonds donated by Lys68 are indicated by yellow chopped tubes, and the hinge region is denoted in magenta. For clarity, only ring atoms of each ligand are shown. Please note that ligand locations in the selected ensemble of template structures sample the whole ATP-binding site.

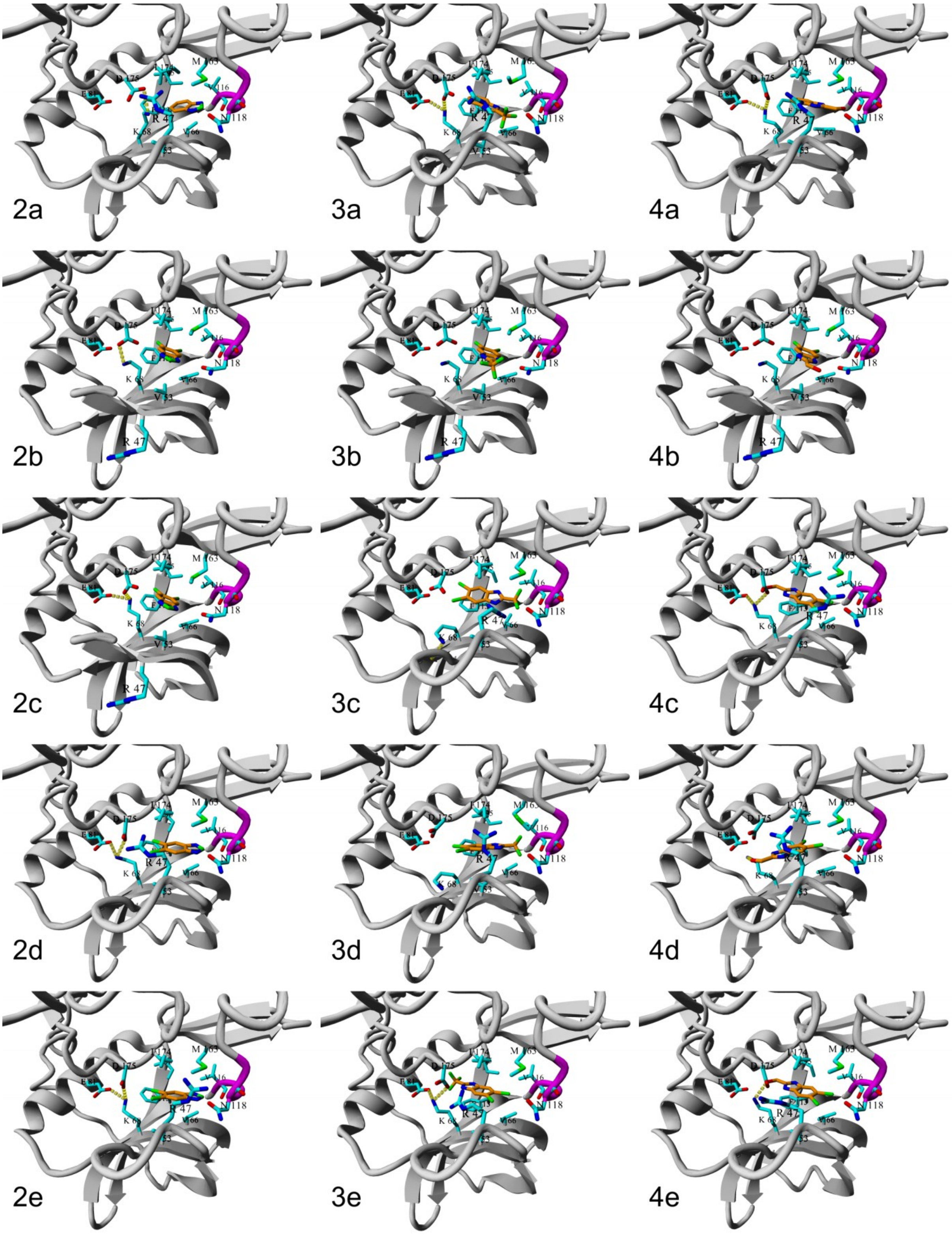

Figure S40. Orientation of **2a-e**, **3a-e** and **4a-e** taken from the lowest-energy clusters obtained with the aid of VINA-Autodock. All residues directly involved in ligand binding are shown in stick representation, the hinge region is in magenta.

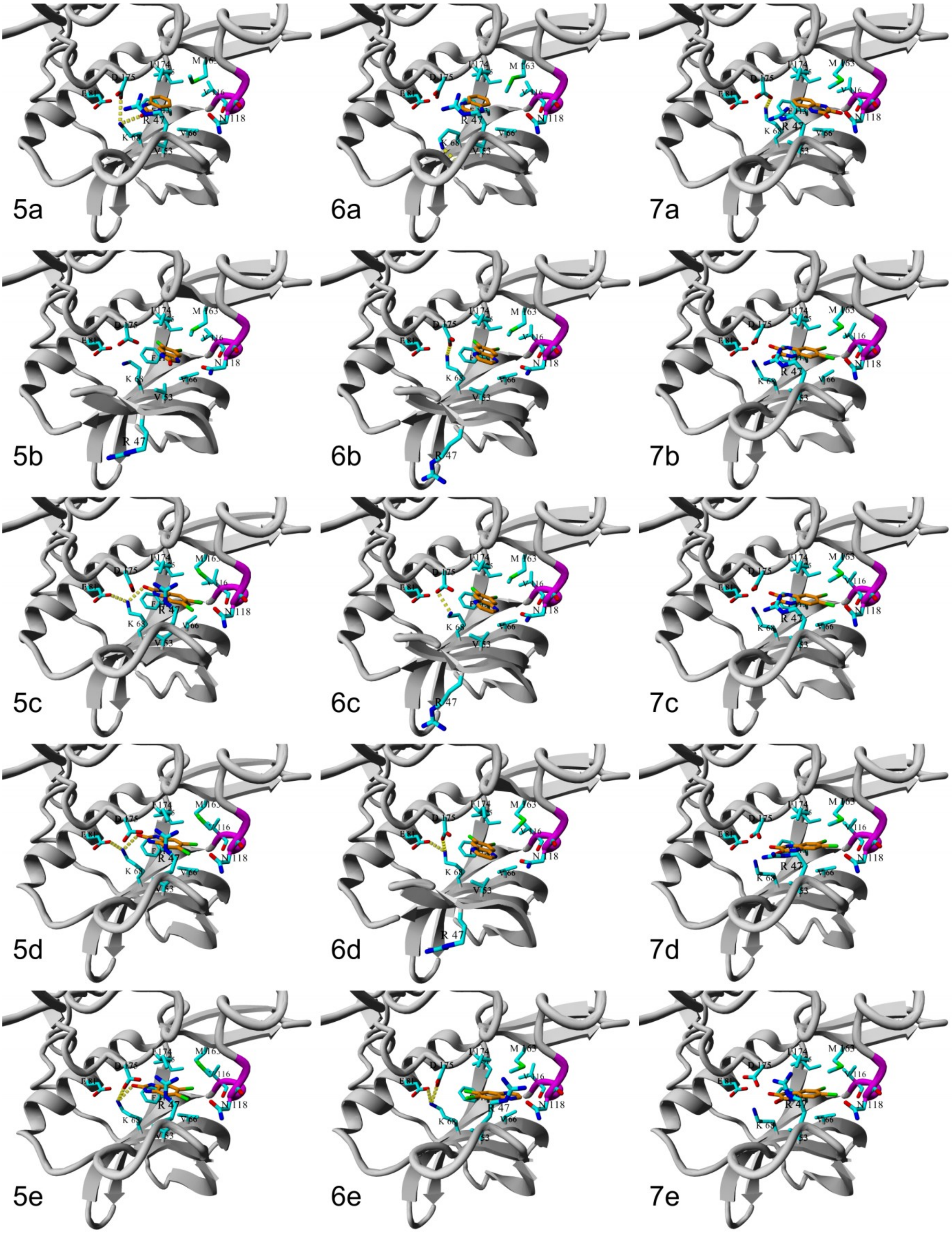

Figure S41. Orientation of **5a-e**, **6a-e** and **7a-e** taken from the lowest-energy clusters obtained with the aid of VINA-Autodock. All residues directly involved in ligand binding are shown in stick representation, the hinge region is in magenta.
